# Teaching and assessing communication skills in the postgraduate medical setting: a systematic scoping review

**DOI:** 10.1186/s12909-021-02892-5

**Published:** 2021-09-09

**Authors:** Xiu Hui Tan, Malia Alexandra Foo, Shaun Li He Lim, Marie Bernadette Xin Yi Lim, Annelissa Mien Chew Chin, Jamie Zhou, Min Chiam, Lalit Kumar Radha Krishna

**Affiliations:** 1 Yong Loo Lin School of Medicine, National University of Singapore, 11 Hospital Dr, Singapore, 169610 Singapore; 2 Division of Supportive and Palliative Care, National Cancer Centre Singapore, 11 Hospital Crescent, Singapore, 169610 Singapore; 3 Medical Library, National University of Singapore Libraries, Block MD 6, 14 Medical Drive, #05-01, Singapore, 117599 Singapore; 4 Lien Centre of Palliative Care, Duke-NUS Graduate Medical School, 8College Road, Singapore, 169857 Singapore; 5 Duke-NUS Medical School, National University of Singapore, 8 College Rd, Singapore, 169857 Singapore; 6 Division of Cancer Education, National Cancer Centre Singapore, 11 Hospital Crescent, Singapore, 169610 Singapore; 7 Palliative Care Institute Liverpool, Academic Palliative & End of Life Care Centre, Cancer Research Centre, University of Liverpool, 200 London Rd, Liverpool, L3 9TA UK; 8 Centre of Biomedical Ethics, National University of Singapore, Block MD 11, 10 Medical Drive, #02-03, Singapore, 117597 Singapore; 9 PalC, The Palliative Care Centre for Excellence in Research and Education, PalC c/o Dover Park Hospice, 10 Jalan Tan Tock Seng, Singapore, 308436 Singapore

**Keywords:** Communication, Skills training, Teaching, Assessment, Medical education, Postgraduate

## Abstract

**Background:**

Poor communication skills can potentially compromise patient care. However, as communication skills training (CST) programs are not seen as a priority to many clinical departments, there is a discernible absence of a standardised, recommended framework for these programs to be built upon. This systematic scoping review (SSR) aims to gather prevailing data on existing CSTs to identify key factors in teaching and assessing communication skills in the postgraduate medical setting.

**Methods:**

Independent searches across seven bibliographic databases (PubMed, PsycINFO, EMBASE, ERIC, CINAHL, Scopus and Google Scholar) were carried out. Krishna’s Systematic Evidence-Based Approach (SEBA) was used to guide concurrent thematic and content analysis of the data. The themes and categories identified were compared and combined where possible in keeping with this approach and then compared with the tabulated summaries of the included articles.

**Results:**

Twenty-five thousand eight hundred ninety-four abstracts were identified, and 151 articles were included and analysed. The Split Approach revealed similar categories and themes: curriculum design, teaching methods, curriculum content, assessment methods, integration into curriculum, and facilitators and barriers to CST.

Amidst a wide variety of curricula designs, efforts to develop the requisite knowledge, skills and attitudes set out by the ACGME current teaching and assessment methods in CST maybe categorised into didactic and interactive methods and assessed along Kirkpatrick’s Four Levels of Learning Evaluation.

**Conclusions:**

A major flaw in existing CSTs is a lack of curriculum structure, focus and standardisation. Based upon the findings and current design principles identified in this SSR in SEBA, we forward a stepwise approach to designing CST programs. These involve 1) defining goals and learning objectives, 2) identifying target population and ideal characteristics, 3) determining curriculum structure, 4) ensuring adequate resources and mitigating barriers, 5) determining curriculum content, and 6) assessing learners and adopting quality improvement processes.

**Supplementary Information:**

The online version contains supplementary material available at 10.1186/s12909-021-02892-5.

## Introduction

Effective doctor-patient communication boosts patient safety and the patient experience [1, 2]. It also improves treatment adherence and reduces malpractice suits and burnout amongst physicians [3, 4]. Whilst the General Medical Council, CanMEDS and the Accreditation Council for Graduate Medical Education (ACGME) [5–8] regard communication skills as a core competency, efforts to advance communication skills training (CST) in medical schools and residency programs remain poorly coordinated [9–14] and, perhaps more concerning, still tethered to the belief that good communications can be learnt ‘on the job’ [15].

With there being a wide array of skills to be mastered, including being able to gather information [15], consider the patient’s circumstances and needs, adapt communication styles and content, facilitate open and respectful discussions and shared decision making, negotiate a personalised patient-centred treatment plan, give therapeutic instructions in an empathetic and understandable manner and establish a caring, responsive doctor-patient relationship, the need for a structured CST program for medical students and physicians is evident [16, 17]. In addition, amidst suggestions that communications skills degrade over time, a longitudinal CST program that melds training, clinical experience, assessments, and reflective practice supported by role modelling, coaching and mentoring is critical [18, 19]. Such a longitudinal approach would be consistent with Hoffman et al. [20]‘s recommendation aimed at developing adaptive clinical communication skills that are responsive to the needs of different patients in different circumstances.

However, facing the recalcitrant notion in some quarters that good communications skills are a “an easy and soft science … not worth studying” [21], design and operationalising longitudinal CST programs face significant obstacles. We believe an evidence-based review of prevailing practices and outcomes will be useful in addressing these misconceptions and will help to facilitate the reshaping of attitudes and thinking towards CST.

### Rationale for this review

Acknowledging growing evidence of the impact of CST in medical schools and the influence of different healthcare and education systems, practice settings and practical considerations [22] on the structure and content of CST programs, we focus on better understanding current approaches to CST in the postgraduate setting. The lessons learnt will inform efforts to advance an evidence-based framework for a CST curriculum that may be applied in different countries, and sociocultural settings.

## Methodology

A systematic scoping review (SSR) is proposed to map prevailing practice and clarify concepts, definitions and key characteristics of CST practice in the extant literature so as to guide design of an evidence-based CST program [23–29]. An SSR is also able to identify gaps in prevailing knowledge on CSTs [30, 31]. Rooted in Constructivist ontology and Relativist epistemology, SSRs are well suited for considering the effects of clinical, academic, personal, research, professional, ethical, psychosocial, emotional, legal, and educational settings and learning environment upon CST processes [32–38]. Here, a Relativist lens captures the impact of the learner’s various CST training experiences which Positivist and Post-Positivist approaches fail to consider. However, whilst these considerations present SSRs as the preferred approach to scrutinising the width, depth, and longitudinal effects of CST, SSRs continue to suffer from a lack of a structured approach that compromises its trustworthiness and reproducibility.

**Fig. 1:**
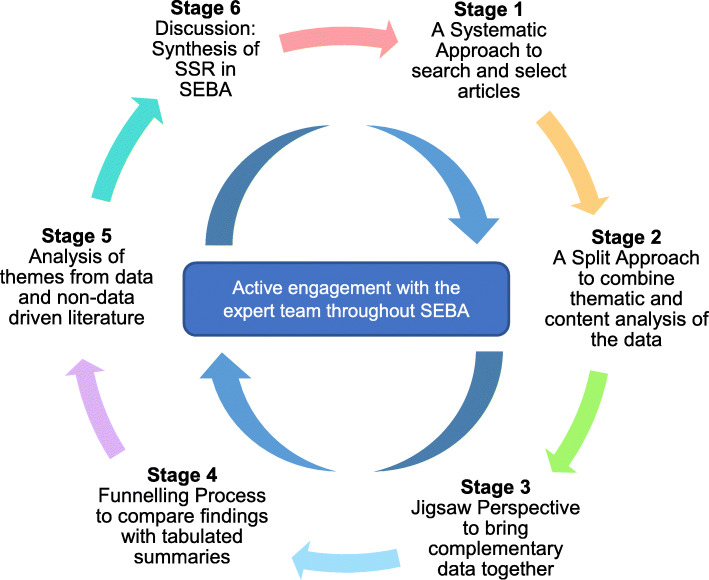
The SEBA process

To overcome these problems facing SSRs, we adopt Krishna’s Systematic Evidence-Based Approach (SEBA) (henceforth SSR in SEBA) [31, 39, 40]. SSRs in SEBA are shown to be well suited to review various aspects of medical education [23, 24, 41–53]. By employing SEBA’s Systematic Approach, Split Approach, Jigsaw Perspective, Funnelling Process, Analysis of Data and Non-Data Driven Literature, and SSR Synthesis (Fig. 1), this SSR in SEBA will provide a holistic picture of CST programs [54–58].

To ensure accountability, transparency and reproducibility, an expert team involving medical librarians from the Yong Loo Lin School of Medicine (YLLSoM) at the National University of Singapore and the National Cancer Centre Singapore (NCCS) and local educational experts and clinicians from NCCS, the Palliative Care Institute Liverpool, YLLSoM and Duke-NUS Medical School (henceforth the expert team) will be consulted at each stage of the SEBA methodology [59–62].

### Stage 1: systematic approach

A. *Determining the title and background of review*

Focusing on CSTs in the postgraduate medical setting, the research and expert teams set out the overall objectives of the SSR in SEBA and determined the population, context and concept to be evaluated.

B. *Identifying the research question*

The primary question was determined to be: *“what is known of prevailing approaches to communication skills training in the postgraduate medical setting?”*

C. *Inclusion Criteria*

A Population, Intervention, Comparison and Outcome (PICOS) format, outlined in Table 1, was adopted to guide the research process [63, 64]. To ensure a sustainable review and to remain focused upon general communication skills used in verbal and non-verbal communications between physicians and patients, we did not include articles focusing on interprofessional communication in this review given its distinct role in training, and in order to accommodate to existing manpower and time constraints [65]. However, articles with a minor focus on interprofessional communication were still included and analysed if their main focus was on physician-patient communication.

D. *Identifying relevant studies*

**Table 1:** PICOS, inclusion and exclusion criteria applied to database search

PICOS	Inclusion Criteria	Exclusion Criteria
**Population**	• Postgraduate doctor or physician or resident, medical officer, registrar, house officer, attending, consultant • Doctor-patient communications • Hospital setting • English Language	• Undergraduate medical students • Veterinary science or Dentistry or Nursing • Allied health specialties such as Pharmacy, Dietetics, Physiotherapy, Podiatry, Occupational Therapy
**Intervention**	• Training of doctors or physicians or residents to improve physician-patient communications • Assessment of doctors or physicians or residents on physician-patient communication skills • Curriculum on doctor-patient communications, including approaches, content and assessment methods	• Interprofessional communications
**Comparison**	• Various forms of curriculum initiatives to improve communication skills • Prevailing theories and principles that guide current teaching methods • Assessment methods and domains of communication skills	
**Outcome**	• To incorporate effective communication training in a new communications curriculum, or to improve existing programs for postgraduate medical training	
**Study design**	• Articles published from 1st January 2000 to 31st December 2020 • Published in English Language • Databases: PsycINFO, EMBASE, PubMed, ERIC, CINAHL, Scopus and Google Scholar • Mixed-methods research, meta-analyses, systematic reviews, randomised controlled trials, cohort studies, case-control studies, cross-sectional studies and descriptive papers • Grey Literature, electronic and print information not by commercial publishing • Case reports and series, ideas, editorials, perspectives, and conference abstracts	

Guided by the expert team and prevailing descriptions of CST programs, the research team developed the search strategy for the PubMed, Embase, PsycINFO, ERIC, Scopus, CINAHL, Google Scholar databases. The full PubMed search strategy may be found in Additional file 1: Appendix 1. Independent searches were carried out through the seven databases. All research methodologies (quantitative and qualitative) in articles published or translated into English were included. To accommodate existing manpower and time constraints, the search was confined to articles published between 1st January 2000 and 31st December 2020 [65]. Additional hand searching of seven leading journals in medical education (Academic Medicine, Medical Education, Medical Teacher, Advances Health Sciences Education, BMC Medical Education, Teaching and Learning in Medicine and Perspectives on Medical Education) was conducted to ensure key articles were included. To cover further ground, the references of the included articles obtained from the above methods were screened to further include relevant articles.

E. *Selecting studies to be included in the review*

Six members of the research team independently reviewed all identified titles and abstracts, created individual lists of titles to be included and discussed these online. Sandelowski and Barroso [66]‘s ‘negotiated consensual validation’ was used to achieve consensus on the final list of titles to be reviewed. Here, ‘negotiated consensual validation’ refers to

*“a social process and goal, especially relevant to collaborative, methodological, and integration research, whereby research team members articulate, defend, and persuade others of the “cogency” or “incisiveness” of their points of view or show their willingness to abandon views that are no longer tenable. The essence of negotiated validity is consensus”*. (p.229).

Scrutinising the final list of titles to be reviewed, the research team independently downloaded all the full text articles on the final list of titles, studied these, created their own lists of articles to be included and discussed their findings online at research meetings. ‘Negotiated consensual validation’ was used to achieve consensus on the final list of articles to be analysed.

### Stage 2: split approach

To enhance the trustworthiness of the review, a Split Approach was employed [67, 68]. This entailed concurrent analysis of the included data using Braun and Clarke [69]‘s approach to thematic analysis and Hsieh and Shannon [70]‘s approach to directed content analysis by two independent groups of at least three reviewers.

A. *Thematic Analysis*

Three members of the research team employed thematic analysis to independently identify key aspects of CST programs across various learning settings, goals, learner and tutor populations [71–79]. This approach was adopted as it helped to circumnavigate the wide range of research methodologies present amongst the included articles preventing the use of statistical pooling and analysis [80, 81].

A reiterative step-by-step analysis was carried out in which codes were constructed from the explicit surface meaning of the text [82]. In Phase 1, the research team carried out independent reviews and ‘actively’ reading the included articles to find meaning and patterns in the data [82–86]. In Phase 2, codes were collated into a code book to code the rest of the articles. As new codes emerged, these were associated with previous codes and concepts to create subthemes. In Phase 3, the subthemes were organised into themes that best depicted the data. An inductive approach allowed themes to be “defined from the raw data without any predetermined classification” [86]. In Phase 4, the themes were refined to best represent the whole data set. In Phase 5, the research team discussed the results of their independent analysis online and at reviewer meetings. Negotiated consensual validation was used to determine the final list of themes.

B. *Directed Content Analysis*

Concurrently, three members of the research team employed directed content analysis to independently review all the articles on the final list. This involved “identifying and operationalising a priori coding categories” by classifying text of similar meaning into categories drawn from prevailing theories [87–91]. In keeping with SEBA’s pursuit of an evidence-based approach, the research team selected and extracted codes and categories from Roze des Ordons (2017)‘s article entitled *“From Communication Skills to Skillful Communication: A Longitudinal Integrated Curriculum for Critical Care Medicine Fellows”* [92]. Use of an evidence-based paradigm article to extract codes from was also in line with SEBA’s goal of ensuring that the review is guided by practical, clinically relevant and applicable data.

In keeping with deductive category application, coding categories were reviewed and revised as required. The research team discussed their findings online to achieve consensus.

#### Quality assessment of studies

To enhance methodological rigour and to provide reviewers with a chance to evaluate the credibility of the conclusions and the transferability of the findings, two research members carried out individual appraisals of the included quantitative studies using the Medical Education Research Study Quality Instrument (MERSQI) [93] and of the included qualitative studies using the Consolidated Criteria for Reporting Qualitative Studies (COREQ) [94]. The summary of the quality assessments may be found in Additional file 2: Appendix 2 as well.

### Stage 3. jigsaw perspective

The themes and categories from the Split Approach are viewed as pieces of a jigsaw puzzle where areas of overlap allow complementary pieces to be combined. These are referred to as themes/categories.

To create themes/categories, the Jigsaw Perspective referenced Phases 4 to 6 of France et al. (2019) [95]‘s adaptation of Noblit and Hare (1998) [96]‘s seven phases of meta-ethnography. As per Phase 4, the themes and the categories identified are grouped according to their focus. These groups are contextualised by reviewing the articles from which the themes and categories were drawn from. This process is facilitated by comparing the findings with tabulated summaries of the included articles that were created in keeping with recommendations drawn from Wong, Greenhalgh [97]‘s *“RAMESES publication standards: meta-narrative reviews”* and Popay, Roberts [98]‘s *“Guidance on the conduct of narrative synthesis in systematic reviews”*.

In keeping with France et al’s adaptation, reciprocal translation was used to determine if the themes and categories could be used interchangeably. This allowed the themes and categories to be combined to form themes/categories.

### Stage 4: funnelling process

The funnelling process saw the themes/categories juxtaposed with key messages identified in the tabulated summaries (Additional file 1: Appendix 2), and reciprocal translation was used to determine if they truly reflected the data. Once verified, the themes/categories formed funnelled domains and served as the ‘*line of argument’* in the discussion synthesis of the SSR in SEBA (Stage 6).

## Results

**Fig. 2:**
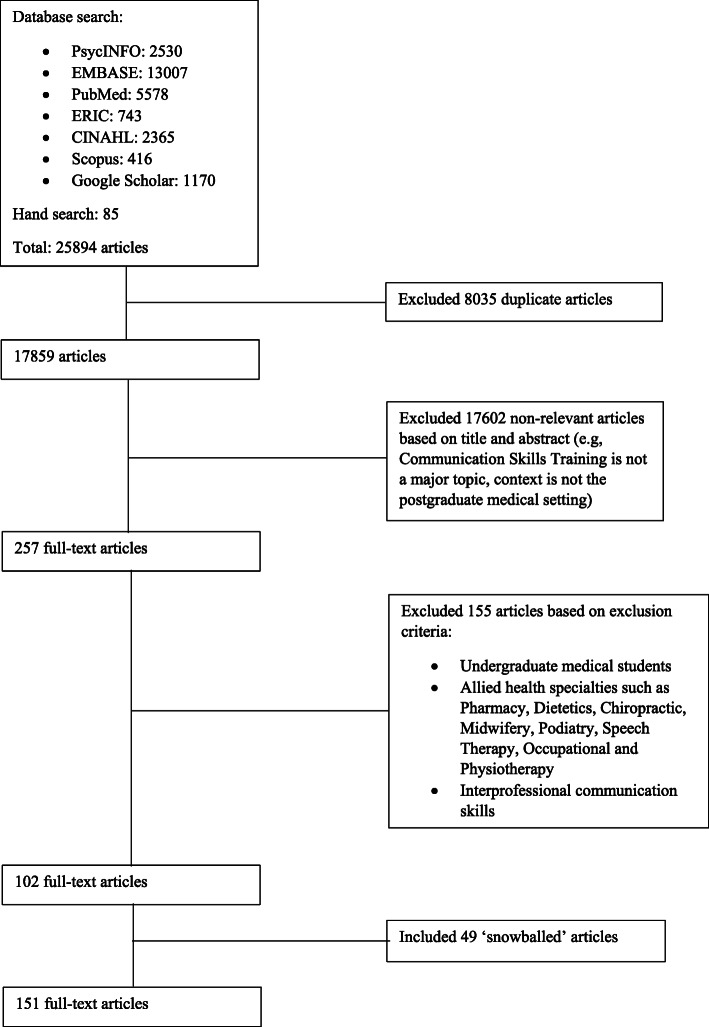
PRISMA Flowchart

Twenty-five thousand eight hundred ninety-four abstracts were identified, 257 full-text articles were reviewed, and 102 full-text articles were included. ‘Snowballing’ of references from these included articles saw a further 49 full-text articles added and analysed, bringing the total number to 151 (Fig. 2). The Split Approach revealed similar themes and categories allowing the Jigsaw Perspective to forward six themes/categories and the Funnelling Process to forward six funnelled domains: curriculum design, teaching methods, curriculum content, assessment methods, integration into curriculum, and the facilitators and barriers to CST.

For ease of review and given that most of the included articles did not elaborate on many of the domains, the data will be presented in tabulated form.

### Curriculum design

A variety of curricula designs were adopted due to differing curricular and program objectives, support and structure; program duration and scheduling in the learner’s training; learner and tutor availabilities, competencies, experiences and settings; assessment methods; education environment; and healthcare and education systems. The principles and models used to structure current CST programs are collated in Table 2.

**Table 2:** Principles and guiding models

Guiding principles and design models	
• ACGME Competencies - Used by GME Programs to Evaluate their Residents in Training [5–8, 11, 33, 99–101] • Team-based communication ◦ Integrative Care Conference [102] ◦ Principles of Shared Decision Making [103] • Communication Skills ◦ Analytic Model of Communications [104] ◦ Principles of Etiquette Based Communication [32] • Patient Interviewing Frameworks ◦ Instructional Framework [7] ◦ Comskil Conceptual Model- Consultation Components [105, 106] • Learning Frameworks ◦ Kolb’s Model of Experiential Learning [107–109] ◦ Peer Teaching [110] ◦ Skill Based Approach based on Peter Maguire’s Work [111] • Assessment Frameworks ◦ Miller’s Pyramid for Assessment of Clinical Competence [107] • Curriculum Development Frameworks ◦ Kern’s Model for Curriculum Development [92] • Situation Specific Guidelines ◦ SPIKES [101, 112, 113] ◦ Existing Guidelines from American Academy of Neurology and European Federation of Neurological Societies (EFNS) for Disclosure of Diagnosis [112] • General Skills ◦ Amenable Communication Skills [114] ◦ Activity Theory and Transformative Learning Theory [115]	

### Teaching methods

Methods to teaching communications may be categorised into didactic and interactive methods. Didactic methods include lectures [3, 13, 116–123], seminars [5, 37], presentations [35, 92, 103, 105, 114, 124–128] and are increasingly hosted on video and online platforms [14, 22, 119, 129, 130]. They are occasionally supplemented by reading material [3, 119, 120].

Interactive methods include role-play with feedback sessions [3, 11, 13, 14, 17, 92, 103–105, 108, 118, 120, 121, 124, 127, 129–136], facilitated workshops [5, 103, 107, 118, 137–142] and group discussions [6, 13, 37, 92, 111, 121, 127, 130, 137, 143, 144]. Interactive methods are also used to facilitate self-directed learning such as facilitator-independent role-play [139, 142, 145–148] where participants may choose to rotate amongst themselves through the roles of patient, physician, observer and critic [149]. They also include independently-held group discussions [7, 16, 35, 102, 141, 142, 150, 151] which encourage learners to learn from their peers through observation [130, 131, 142] and feedback [35, 110, 130, 152, 153], as well as engage in introspective reflection on the role and importance of good communication [5, 6, 8, 16, 103, 108, 125, 126, 142, 146, 150, 151, 153–155]. Feedback and reflective practice [156–158] are increasingly seen as key teaching tools critical to developing adaptive, patient-centred communication, shared decision making and negotiated treatment plans [5, 6, 8, 16, 103, 108, 125, 126, 142, 146, 150, 151, 153–155].

### Content of curriculum

There are a diverse range of topics within current communications curricula. To remain focused upon communications training between patient and physician, we align our findings with the *‘ACGME Core Competencies: Interpersonal and Communication Skills’* [159] as seen in Table 3.

**Table 3:** Content of curriculum

Sub-competency	Elaboration
Create and sustain a therapeutic relationship with patients and families	Structuring the consultation • Opening the discussion by setting the agenda and expectations [16, 22, 126, 160–162] • Utilising simple, clear language and effective questioning skills [17, 102, 114, 163, 164] to gather information [37, 119, 126, 131, 161] • Sharing information effectively [131, 161, 165] • Checking patient understanding [165, 166] • Shared decision making [103, 126, 129, 149, 160, 162, 165, 167] • Providing closure to consult [126, 161, 162] • Summarising [22] Building the physician-patient relationship • Making patient/patient’s family feel at ease [153, 168, 169] • Showing empathy [36, 37, 102, 111, 114, 138, 152, 153, 164, 166, 167, 170, 171] • Showing respect [102–104] • Convey understanding of concerns [153] • Understanding the patient’s perspective [102, 126, 134, 153, 162, 164–166, 169, 171, 172] • Eliciting patient’s wishes, needs, concerns and expectations [16, 163, 170, 172, 173] • Identifying patient's health literacy levels [174] • Motivational interviewing and counselling [37, 116, 140, 165, 174–176] • Employing verbal and non-verbal skills [22, 134, 138, 163, 164, 173] • Listening skills [17, 102, 138, 147, 163, 164, 171] • Non-judgmental communication [147] • Managing patients’ emotions [3, 11, 17, 22, 118, 129, 138, 160, 173, 177] • Culturally and linguistically appropriate communication [99, 102, 140, 147, 152, 163, 171] • How to interact when patient’s relatives are present [118] • How to communicate with patient indirectly through interpreters [36, 175] or over telephone consultations [34] • Communication with adolescents [34] • Communicating with 'difficult' patients or family members [151, 163] or emotional patients [34, 111, 114, 146, 162, 175] • Handling family conflict [153] • Dealing with mismatched expectations [16] • Conducting family discussions [149, 178] • Communication clarity [163] Context-specific skills • End-of-life communication ◦ Using the word ‘dying’ [125] ◦ Conducting goals of care and advance care planning conversations [16, 92, 124, 171, 177, 179–181] ◦ Discussing pain management [180] ◦ Eliciting Do Not Resuscitate orders [109, 182] ◦ Responding to euthanasia requests [16] ◦ Sharing prognostic information with patients [16, 105, 107, 114, 129] ◦ Preparing for death [16, 129] ◦ Managing patient’s reactions [139, 177, 183] ◦ Maintaining patient's welfare [183] ◦ Supporting patient’s decision [179] ◦ Offering organ donation [92] ◦ Pronouncing death [5] • Difficult conversations with seriously ill patients [13, 184] ◦ Explaining a patient’s worsening condition [153] ◦ Explaining that treatments are not indicated [36, 153] ◦ Discussing whether to forego life-sustaining treatment [124] ◦ Transitioning to palliative care [3, 36, 114, 133, 149, 153] • Breaking Bad News [3, 6, 7, 11, 12, 32, 36, 108, 113–115, 118, 119, 124, 127, 129–131, 133, 139, 141, 146, 149, 151–153, 162, 163, 170, 173, 175, 178, 185–188] ◦ Utilising the SPIKES framework [34, 92, 115, 116, 121, 141, 142, 152, 179, 188] ◦ Disclosure of medical complications [6, 32, 137] • Navigating situations with ethical issues [131] • Disclosure of medical errors and apology [36, 92, 99, 127, 151, 189] • Discussing risks/benefits of procedures and obtaining informed consent [6, 119, 146, 151, 165, 170, 186] • New medication and discharge counselling [99]
Work effectively as a member or leader of a health care team	• Managing disagreements between colleagues [111] • Working with ‘difficult’ colleagues [163] • Oral presentations and giving feedback [7, 190, 191] • Leadership skills [36, 153] • Interprofessional communication [7, 99, 111, 190–193] • Writing skills, especially for documentation [194] • Persuasive communication [176] • Reporting findings in a letter to the general practitioner [169]

Amongst this diverse array of topics, there are a few that appear more commonly within particular specialities. These are featured in Table 4.

**Table 4:** Content of curriculum by specialties

Communication skill	Specialty				
	Internal Medicine	Family Medicine	Surgery	Oncology	Others (including Radiology, Obstetrics & Gynaecology, Paediatrics, Anaesthesia, Accident & Emergency, Trauma)
1. **Create and sustain a therapeutic relationship with patients and families**					
a. **Structuring the consultation**					
Opening the discussion by setting the agenda and expectations	[126]	[22]			[16, 160–162]
Utilising simple, clear language and effective questioning skills to gather information	[37, 126]		[119]	[114]	[17, 102, 131, 161, 163, 164]
Sharing information effectively		[165]			[131, 161]
Checking patient understanding		[165, 166]			
Shared decision making	[103, 126]	[165, 167]		[129, 149]	[160, 162]
Providing closure to consult	[126]				[161, 162]
Summarising		[22]			
b. **Building the physician-patient relationship**					
Making patient/patient’s family feel at ease					[153, 168, 169]
Showing empathy	[37]	[166, 167]	[36, 111]	[114, 152]	[102, 138, 153, 164, 170, 171]
Showing respect	[103]				[102, 104]
Convey understanding of concerns					[153]
Understanding the patient’s perspective	[126, 172]	[134, 165, 166]			[102, 153, 162, 164, 169, 171]
Eliciting patient’s wishes, needs, concerns and expectations	[172]			[173]	[16, 163, 170]
Identifying patient’s health literacy levels		[174]			
Motivational interviewing and counselling	[37]	[140, 165, 174, 175]		[116]	[176]
Employing verbal and non-verbal skills		[22, 134]		[173]	[138, 163, 164]
Listening skills					[17, 102, 138, 147, 163, 164, 171]
Non-judgmental communication					[147]
Managing patients’ emotions	[11, 177]	[22]		[3, 118, 129, 173]	[17, 138, 160]
Culturally and linguistically appropriate communication	[99]	[140]		[152]	[102, 147, 163, 171]
How to interact when patient’s relatives are present				[118]	
How to communicate with patient indirectly through interpreters or over telephone consultations		[175]	[36]		[34]
Communication with adolescents					[34]
Communicating with 'difficult' patients or family members or emotional patients	[146, 151]	[175]	[111, 146]	[114]	[34, 146, 162, 163]
Handling family conflict					[153]
Dealing with mismatched expectations					[16]
Conducting family discussions	[178]	[178]		[149]	
Communication clarity					[163]
c. **Context-specific skills**					
i. **End-of-life communication**					
Using the word ‘dying’	[125]				
Conducting goals of care and advance care planning conversations	[177, 179, 180]	[181]			[16, 92, 124, 171]
Discussing pain management	[180]				
Eliciting Do Not Resuscitate orders				[182]	[109]
Responding to euthanasia requests					[16]
Sharing prognostic information with patients	[107]			[114, 129]	[16, 105]
Preparing for death				[129]	[16]
Managing patient’s reactions	[177]			[139]	[183]
Maintaining patient's welfare					[183]
Supporting patient’s decision	[179]				
Offering organ donation					[92]
Pronouncing death		[5]			
ii. **Difficult conversations with seriously ill patients**					
Explaining a patient’s worsening condition					[153]
Explaining that treatments are not indicated			[36]		[153]
Discussing whether to forego life-sustaining treatment					[124]
Transitioning to palliative care			[36]	[3, 114, 149]	[133, 153]
iii. **Other contexts**					
Breaking bad news	[11, 146, 151, 178]	[130, 175, 178]	[12, 32, 36, 119, 127, 146, 186]	[3, 114, 115, 118, 129, 139, 149, 152, 173, 187]	[6, 7, 108, 113, 124, 131, 133, 141, 146, 153, 162, 163, 170, 185, 188]
Disclosure of medical complications			[32, 137]		[6]
Navigating situations with ethical issues					[131]
Disclosure of medical errors and apology	[99, 151, 189]		[36, 127]		[92]
Discussing risks/benefits of procedures and obtaining informed consent	[146, 151]	[165]	[119, 146, 186]		[6, 146, 170]
New medication and discharge counselling	[99]				
2. **Work effectively as a member or leader of a health care team**					
Managing disagreements between colleagues			[111]		
Working with ‘difficult’ colleagues					[163]
Oral presentations and giving feedback				[191]	[7, 190]
Leadership skills			[36]		[153]
Interprofessional communication	[99, 192, 193]		[111]	[191]	[7, 190]
Writing skills, especially for documentation					[194]
Persuasive communication					[176]
Reporting findings in a letter to the general practitioner					[169]

### Assessment methods

There are a variety of assessments methods used to evaluate communication skills. In most of the included articles, details as to when and how these tools are employed were not elaborated upon. Available information is collated in Table 5.

**Table 5:** Criteria in measuring the physician’s communication behaviour

Aspect	Elaboration
Cognitive	• Verbal skills ◦ Clarity of physician’s explanations and, in turn, patient’s understanding [32, 36, 166, 173] ◦ Use of jargon [6, 36, 142, 145] ◦ Encouraging questions [6, 36, 142] • Non-verbal skills ◦ Non-verbal cues [9, 32, 137, 138, 145, 162, 171, 173] ◦ Listening skills [9, 32, 138, 171, 173] • Overall efficacy ◦ Addressing issues, concerns, barriers, and facilitators to medication taking [32, 138, 140, 173] ◦ Patient education competency [155] ◦ Time management [145] ◦ Patient centeredness [32, 37, 102, 104, 118, 171, 175] ◦ Ensuring adequate support [34, 142] ◦ Planning [32, 142, 175]
Affective	• Patient specific ◦ Satisfaction with the consultation [34, 111, 120, 152, 153, 160, 173, 174] ◦ Patient distress [118] ◦ Complaints against the doctor [138] • Physician-patient relationship ◦ Patient’s perceptions of the relationship [143, 152, 155, 173] ◦ Rapport building [22, 35, 104, 162]
Physician attributes	• Professionalism [32, 138, 145, 173, 190–193, 195] • Physical examination [138, 173, 196–198] • Empathy [33, 34, 112, 138, 141, 171, 196] • Compassion [102, 141, 145] • Respect [32, 138, 171, 173] • Individualised attention [32, 138, 173]

Acknowledging the premise that communication skills develop over time and with experience, practice and reflection, it is increasingly necessary to design assessments at the appropriate stage of the learner's development and setting. These assessment methods may be mapped according to the progressive levels of Kirkpatrick’s Four Levels of Learning Evaluation (Table 6) [143].

**Table 6:** Assessment methods and outcome measures

Kirkpatrick Levels	Outcome measured	Assessment method
Level 1: *Participation in training*	• Usefulness of the course [6, 34, 107, 108, 116, 139, 143, 145, 147, 171, 177, 188, 199–201] • Feedback on course structure and teaching methods [32, 37, 110, 116, 139, 143, 174, 199] • Satisfaction with the course [16, 131, 139, 143, 177, 179, 184, 201]	• Post-course survey [6, 16, 37, 108, 129, 131, 147, 167, 168, 178, 188, 189, 201] ◦ Using a Likert scale [6, 34, 107, 110, 116, 139, 143, 171, 177–179, 184] • Focus group session [37]
Level 2a: *Attitudes and perceptions*	• Attitude and perceived importance towards patient-physician communication [16, 35, 99, 103, 126, 139, 146, 153, 161] • Attitude towards communication skills training [36] • Attitude on applying the skills learnt to regular practice [105] • Self-rated confidence in own communication skills [6, 7, 13, 16, 34–37, 103–105, 111, 116, 124–126, 128, 139, 141, 143, 145, 152, 177, 179, 184, 188, 199, 202–204] • Stressfulness during communication [16, 116, 151, 188] • Burnout levels [16, 116, 199]	• Pre- and post -course surveys [16, 36, 99, 105, 111, 139, 143, 145, 151, 153, 179, 188, 204] ◦ Post-course survey only [177] ◦ Using a Likert scale [34, 35, 99, 105, 116, 119, 184, 202] • Questionnaire tools [103, 116, 199, 203]
Level 2b: *Knowledge and skill levels*	• Self-rated skill levels [7, 35, 104, 107, 111, 112, 125, 128, 131, 141, 143, 146, 149, 151, 153, 155, 161, 165, 170, 189, 191, 199–201] • Self-rated knowledge level [37, 107, 139, 146] • Knowledge and skill levels as rated by: ◦ Experienced physician or psychologist or faculty staff or communication trainer [11, 13, 22, 32, 103, 104, 112, 141, 145, 154, 179, 189, 192, 195, 198, 200, 204–208] ◦ Research authors [142, 143] ◦ Peers [36, 131] ◦ Simulated patients [12, 36, 131, 137, 145, 149, 163, 179, 186, 189, 203, 204, 208, 209] ◦ Patients and caregivers [140, 141, 160, 170, 173] • Patient satisfaction with doctor’s communication skills [138, 143] ◦ In-role simulated patient (SP) feedback [164] • Physician’s ability to detect and identify emotion ◦ Self-rated [33] ◦ Comparison between physician’s ability to detect patient's distress and patient’s self-reported distress [118] • Communication scenarios that physicians found difficulty in [116] • Content specific skills [99, 126, 137, 152] ◦ Preparedness to break bad news [34, 112, 116, 139, 141, 199, 200] ◦ Addressing end of life matters [35, 105, 109, 122, 125, 175, 178, 179, 184, 200, 204] ◦ Showing empathy [138] ◦ Discussing patient’s spiritual concerns [184] ◦ Health literacy [163]	• Pre- and post-course surveys and quizzes [34, 104, 107, 116, 125, 126, 139, 141, 143, 151, 153, 165, 193, 199] ◦ Post-course quiz only [189, 201] • Structured self-assessments [37, 191] • Clinical vignettes and case scenarios [139, 142] • Debrief and feedback session [36, 150, 155, 175, 209] ◦ Written feedback [9, 32, 114, 195] • Role playing scenarios ◦ Videotaped or audiotaped [32, 103, 104, 112, 114, 136, 141, 142, 163, 175, 179, 193, 199, 203, 205, 207, 210, 211] ◦ Conducted using simulated patients [11, 32, 34, 36, 103, 118, 129, 133, 137, 149, 163, 177, 179, 186, 189, 199, 203–205, 207, 212–214] • Video or audio recording of the physician’s interaction with real patients [9, 114, 118, 120, 134, 173, 178, 211] • Communication skills tools/ checklists [11, 34, 36, 103, 104, 131, 141–143, 145, 160, 173, 177, 189, 202, 206, 207] ◦ Specifically graded by patients [118, 137, 138, 140, 143, 173, 202] ◦ Tools to identify and detect emotion [33]
Level 3: *Change in behaviour*	• Communication with patients and caregivers [153, 200] • Application of communication skill techniques taught [92, 124, 151] ◦ Frequency of skills practice after the course [6, 103, 124, 139, 146, 149, 173] ◦ Commitment to continued practice of the skills learnt [124] ◦ Rated according to a checklist by trained raters [169]	• Surveys [119, 124, 132, 167, 215] • Direct workplace observation [120, 153, 195, 200, 213, 216] ◦ Feedback from an interprofessional clinician and the patient [92] ◦ Unannounced SP visits [165] ◦ Videotaped encounters [169, 217] • Portfolio to record real life scenarios [151] • Role playing scenarios ◦ SP encounter [103, 215] ◦ Annual evaluation with the use of an assessment form [7] • Patient’s change in behaviour [120, 140, 165]
Level 4: *Long term change in performance and effect on patient care*	• Patient’s satisfaction [14, 152, 153, 185, 215] • Post-consultation anxiety [152] • Communication about cancer screening [165] • Self-rated self-efficacy in challenging communication scenarios [124, 202]	• Survey/questionnaires [14, 185, 202] • Patient family surveys and semi structured interviews [153] • Direct observation ◦ Unannounced SP visits [165] ◦ Video-taped encounters [14, 22, 129, 181, 202, 215]

### Integration of training

Most programs were part of a formal residency/ fellowship curriculum which provided ‘protected time’ for teaching [141]. However, these programs varied in duration with some offering CST as a single component of scheduled teachings and grand rounds, whilst others via a stand-alone communications retreat, workshop or course [11, 12, 119, 125, 126, 135, 137, 146, 147, 185, 208]. These ad-hoc sessions were often more flexible [114, 149, 173] to accommodate to the busy schedules of the physicians [143]. They tended to be shorter in duration, ranging from 1 to 3 hours per session and focused on the specific needs of the learner population [16, 111, 149, 167].

Others spanned several sessions [3, 6, 13, 14, 35, 37, 99, 116, 123, 124, 131, 139, 140, 199, 202, 212, 218]. These longitudinal sessions were often structured in a step-wise manner, with the intent of first delivering key knowledge and developing requisite skills before more complicated topics are introduced [22, 36, 92, 117, 127], highlighting vertical integration within the spiralled curriculum. These multiple sessions often take place during specific rotations at regular intervals [13, 17, 35, 103, 107, 121, 130, 142, 153, 174, 175] over several months [7, 33, 100, 104, 118, 128, 133, 134, 150, 151, 160, 165]. For example, Ungar et al. [130] implemented a 14-session ‘breaking bad news’ training program for second-year family medicine residents where core skills in acknowledging patient needs were first taught, followed by techniques on breaking bad news and confronting distressing questions. Newcomb et al. [127] used a similar spiralled curriculum that extended over a two-year period to teach communication skills to surgical residents with more advanced topics such as crisis management coming in at a later stage.

### Resources, facilitators and barriers to CST

- Resources

Resources required to establish and sustain CST programs are summarised in Table 7.

**Table 7:** Resources for a sustainable curriculum

Resources required	Elaboration
Human resources	• Course Coordinators [13, 92, 155] • Course Facilitators ◦ Faculty Instructors [37, 122, 135, 148] ◦ Multidisciplinary teams [13, 102, 116, 125, 128] • Course Reviewers [92, 104, 127, 131, 140, 141, 199, 200] • Standardized Patients ◦ Peers [145, 151] ◦ Former Patients [12, 36] ◦ Volunteers [36, 127, 132] • Actors/Actresses [16, 103, 109, 124, 142, 186, 219]
Financial resources	• Remuneration for course facilitators [8, 92, 108, 165] • Remuneration for course reviewers [150] • Remuneration for Simulated Patients [165]

b. Facilitators

Facilitators are factors that aid effective delivery and reception of CST. These include faculty support [37, 92, 108, 127], opportunities to attend courses [151], a platform for feedback [92], faculty training [105, 116, 124, 129, 132, 133, 153, 185, 199, 200, 208] and simulation sessions [13, 119, 135, 186].

c. Barriers

Barriers impede CST programs. These barriers include curriculum factors, physician factors and patient factors. Curriculum factors include the lack of protected time [35, 37, 117, 119, 125, 127, 146, 147, 149, 153, 170, 173–175], logistical and manpower constraints [14, 32, 100, 139, 142, 145, 146, 150], inadequate resources [150], inadequate faculty support [37, 117] and a lack of buy in from participants and colleagues [92].

Physician factors include overcoming complacency with regards to CST [16, 99, 175, 202], overemphasis on technical aspects of clinical practice over soft skills [36, 102, 124, 150, 173] and difficulty measuring communication-related performance indices [8, 171].

Patient factors encompass both simulated patients and real patients. Simulated patients have to be recruited, trained and remunerated [14, 120, 131, 145]. Employ of former patients acting as simulated patients creates concerns over their biases and wellbeing [12, 36]. Limitations of having staff or peers take on the role of simulated patient lie in their variable acting skills and their ability to convey the gravity of the situation and the integrity of the encounter [12]. On the other hand, real patients may give little to no criticism to their physicians, hence limiting awareness of areas of improvement [118, 202]. Elderly patients are especially unwilling to disclose their emotional distress thus making it difficult for physicians to pick up social clues [118]. Patients may also mistakenly perceive politeness as having good communication skills [202].

### Stage 5: analysis of evidence and non-evidence-based literature

With quality appraisals highlighting that data taken from grey literature, opinion, perspectives, editorial, letters and other non-primary data-based articles (henceforth non-evidence-based data) were shown to be consistently poor, the expert team determined that the impact of non-evidence-based data upon the discussions and conclusions drawn in the SSR in SEBA should be evaluated.

To do so, the research team carried out separate and independent reviews and thematic analyses of evidence-based data from bibliographic databases and compared them to the themes drawn from non-evidence-based data. The themes from both groups were found to be similar, thus allowing the expert and research teams to conclude that the non-evidence-based data included in this review did not bias the analysis untowardly.

## Discussion

### Stage 6: synthesis of SSR in SEBA

In answering its primary research question, this SSR in SEBA reveals growing employ of designated CST programs within formal curricula. Taking the form of spiralled curricula to support structured and longitudinal programs, many CST programs use a combination of didactic and interactive approaches in tandem with context-dependent tools aimed at assessing the learner’s expected abilities so as to facilitate learner-specific feedback and support. This maturing approach to CST in postgraduate medical education is scaffolded upon horizontal and vertical integration of communications training that sees CST training sessions carried out at a point where particular topics are especially relevant to the learner, highlighting greater education theory grounded approaches in their design [127, 130]. This underlines the rationale for different contents being inculcated in different settings.

Efforts at curricula design of CST programs, too, have taken a more holistic perspective with programs being framed by clearly delineated design models, frameworks and/or guiding principles. This may involve use of situation-specific guidelines such as SPIKES [101, 112, 113]. Yet, this approach also pays due consideration to the setting and relevance of the content in order to motivate the learner to actively participate in a CST session that activates their prevailing knowledge and skills. Use of Knowles’s Adult Learning Theory and its latter reiterations such as Taylor and Hamdy [220]‘s Multi-theories Model that include Kolb’s Cycle [221, 222] scaffolded around Miller’s Pyramid have also guided the integration of reflective practice and timely, personalised and appropriate support in formal curricula. Use of formal curricula also helps ensure that structured interactions set the stage for longitudinal development of skills and knowledge, ‘protected time’ for communication teaching, and better blending of didactic sessions with interactive sessions at ward rounds and grand rounds and/or online discussions.

Based upon the findings and current design principles identified in this SSR in SEBA, we forward a stepwise approach to designing CST programs. This is outlined in Table 8.

**Table 8:** Steps to planning a CST curriculum

Steps	Description
1: Define goals and learning objectives	Often based upon a needs assessment, the support mechanisms, support structures, resources and curriculum, as well as defining the overall goals of the program and the target population to be trained, will help shape the learning objectives of the CST, the codes of conduct, roles and responsibilities of learners and tutors, which will help to align expectations and standardise teaching and assessment methods.
2: Identify target population and ideal characteristics	Understanding the range of individual goals and competencies amongst participants, where they are in their learning journeys, their roles in their particular speciality, the specific kinds of cases that they will face, and the level of competency that should be expected of them will also inform the design of the program and curation of topics to be taught.
3: Determine the curriculum structure	Realising a longitudinal, structured [5, 32, 116, 117, 124, 137, 139, 142, 146, 150–152, 160, 175, 179, 184, 200] and spiralled [22, 36, 92, 117, 127] curriculum within whilst taking into account practical considerations and training contexts requires careful thought. Due consideration to horizontal and vertical integration will determine the contents to be taught and the timing of these sessions. Establishment of protected time will also shape curriculum design.
4: Ensure adequate resources and mitigate the barriers	There must be effective and sustainable human and financial resources. This includes trained faculty [105, 116, 124, 153, 199, 200], communication and feedback platforms [92, 104, 131, 140, 141, 199, 200] and simulated patients [16, 36, 103, 124, 142, 145, 151, 219]. Particularly important is effective oversight and support of the program [37, 117, 170, 174].
5: Determine the curriculum content	The CST will comprise of basic communication knowledge and skills revision, followed by the inclusion of more advanced competencies. Basic communication knowledge and skills to be built on • include verbal and non-verbal behaviour, empathy, understanding the patient holistically as a person and providing patient-centred care. • need to be part of a longitudinal [137] and spiralled program that will be reviewed consistently [143] With longitudinal support and assessment learners will also develop deeper skills, reflective learning and scaffolding for advanced skills [92].
6.Assess the learner and adopt quality improvement processes	Learner assessments should be accompanied by evaluation of the program and feedback from all the participants. The impact of the sessions should be evaluated longitudinally, and lessons learnt should be used to improve the program.

Based on the principles set out in Table 8, we believe that this structured framework to the teaching and assessment of CSTs may be used in a variety of contextual and sociocultural settings and fine-tuned to the learner’s knowledge, skills, attitudes and, increasingly, behaviour over time.

The culmination of these finding also brings to the fore several considerations, not least the notion that CST programs should be blended with CST in medical schools so as to deepen and widen the spiralled curriculum. Such an approach would necessitate the use of portfolios to inform learners and tutors of communication gaps, and facilitate reflections, remediation and progress towards the achievement of overarching Entrustable Professional Activities (EPA)s [223, 224]. Changing thinking, attitudes, conduct and practice also alludes to the role of CST in professional identity formation which also warrants further study.

## Limitations

Whilst we have conducted a three-tiered searching strategy, through independent searching of selected databases, repeated sieving of reference lists of the included articles (snowballing) and searching of prominent medical education journals, the usage of specific terms and inclusion of only papers in the English languages may have led to important papers being missed. Similarly, whilst use of the Split Approach and tabulated summaries in SEBA allowed for triangulation and ensured that a holistic perspective was constructed, inherent biases amongst the reviewers would still impact the analysis of the data and construction of themes.

However, we believe that through the employment of SEBA, this review has the required rigour and transparency to render this a reproducible and comprehensive article. We hope that the findings of this systematic scoping review will be of interest to educators and program designers in the postgraduate medical setting and will help to guide the design of successful CST programs to fortify physicians in this essential domain.

## Conclusion

As we look forward to engaging in this exciting and rapidly evolving aspect of medical education and practice, we hope to evaluate our proposed framework in practice in our coming research and focus attention upon the use of portfolios in CST programs. This is particularly in considering the possibility that CST may have a hand in shaping professional identity formation. Further understanding of theories and approaches underpinning CST use within medical training is in need of further study as is the role of online multimedia platforms and the medical humanities in teaching adaptive, empathetic and personalised communication skills.

## Supplementary Information

**Additional file 1: Appendix 1.** PubMed Search Strategy.

**Additional file 2: Appendix 2.** Tabulated summaries and quality assessment of included articles.

## Data Availability

All data generated or analysed during this study are included in this published article and its supplementary information files.

## Abbreviations

ACGME: Accreditation Council for Graduate Medical Education
COREQ: Consolidated Criteria for Reporting Qualitative Studies
CST: Communication Skills Training
GME: Graduate Medical Education
MERSQI: Medical Education Research Study Quality Instrument
NCCS: National Cancer Centre Singapore
PICOS: Population, Intervention, Comparison, Outcome, Study design
SEBA: Systematic Evidence-Based Approach
SSR: Systematic Scoping Review
YLLSoM: Yong Loo Lin School of Medicine
