# Supplementary material for: Teaching and assessing communication skills in the postgraduate medical setting: a systematic scoping review
Source: BMC Med Educ. 2021 Sep 9;21:483. doi: 10.1186/s12909-021-02892-5 (PMC8431930; doi:10.1186/s12909-021-02892-5)
Supplement: Supplementary file 1 — Additional file 1: Appendix 1. PubMed Search Strategy. [file 12909_2021_2892_MOESM1_ESM.docx]

**Teaching and Assessing Communication Skills in the Postgraduate Medical Setting: A Systematic Scoping Review**

**Supplementary Material**

*Appendix 1: PubMed Search Strategy*

| **Number** | **Search logic** | **Search term** |
| --- | --- | --- |
| 1 | Physicians | “Students, Medical”[Mesh] OR (“Students”[Mesh] OR student[tiab] OR students[tiab]) AND ("Physicians"[Mesh] OR doctor[tiab] OR doctors[tiab] OR physician[tiab] OR physicians[tiab] OR clinical[tiab] OR medical[tiab]) |
| 2 | Communication and education | ((“Communication”[Mesh] OR Communication[tiab] OR Communications[tiab] OR Communicating[tiab] OR Communicate[tiab]) AND (“Delivery of Health Care”[Mesh] OR health[tiab] OR healthcare[tiab] OR medical[tiab] OR clinical[tiab] OR medicine[tiab]) AND (“Education”[Mesh] OR educate[tiab] OR education[tiab] OR educating[tiab] OR educations[tiab] OR curricula[tiab] OR curriculum[tiab] OR teaching[tiab] OR teachings[tiab] OR teach[tiab] OR learn[tiab] OR learning[tiab] OR competence[tiab] OR competency[tiab])) |
| 3 | Methods | “Program Evaluation” [Mesh] OR “Teaching/methods"[Mesh] OR “Education/methods"[Mesh] OR Methods [tiab] OR Method [tiab] OR Methodology [tiab] OR Methodological[tiab] OR pedagogies [tiab] OR pedagogy [tiab] |
| 4 | Curriculum | "Teaching Materials"[Mesh] OR “Curriculum”[Mesh:NoExp] OR Competency-Based Education [Mesh] OR “Education/standards"[Mesh] OR Problem-Based Learning [Mesh] OR Content [tiab] OR Contents [tiab] OR educational resource [tiab] OR educational resources [tiab] OR Framework* [tiab] OR Groundwork [tiab] OR Groundworks [tiab] OR Educational System* [tiab] OR  “EPA”[tiab] OR “EPAs”[tiab] OR  entrustable professional activity[tiab]  OR entrustable professional activities[tiab] |
