# Supplementary material for: Teaching and assessing communication skills in the postgraduate medical setting: a systematic scoping review
Source: BMC Med Educ. 2021 Sep 9;21:483. doi: 10.1186/s12909-021-02892-5 (PMC8431930; doi:10.1186/s12909-021-02892-5)
Supplement: Supplementary file 2 — Additional file 2: Appendix 2. Tabulated summaries and quality assessment of included articles. [file 12909_2021_2892_MOESM2_ESM.docx]

**Teaching and Assessing Communication Skills in the Postgraduate Medical Setting:
A Systematic Scoping Review**

**Supplementary Material**

*Appendix 2: Tabulated summaries and quality assessment of included articles*

| **S/N** | **Author, Year, Article Title** | **Type of Study** | **Purpose of study** | **Methodology** | **Study population** | **Results** | **MERSQI score (Max 18)** | **COREQ score (Max 32)** |
| --- | --- | --- | --- | --- | --- | --- | --- | --- |
| 1 | Alexander, S. C., et al. (2006) A controlled trial of a short course to improve residents' communication with patients at the end of life. | Prospective study | High-quality palliative care requires physicians who communicate effectively, yet many do not receive adequate training. Leading efforts to demonstrate the effectiveness of such training have involved time-intensive programs that included primarily attending physicians, which have been conducted outside of the United States. The goal of this study was to evaluate the effect of a short course to improve residents’ communication skills delivering bad news and eliciting patients’ preferences for end-of-life care. | This prospective trial enrolled internal medicine residents at Duke University Medical Center from 1999 to 2001. The course consisted of small-group teaching with lecture, discussion, and role-play. The outcome measure was observed communication skills delivering bad news and eliciting patients’ preferences for end-of-life treatment, assessed via audio-recorded standardized patient encounters before and after receiving the intervention. | Internal Medicine residents | Residents attending the course demonstrated statistically significant increases in their overall skill ratings in the delivery of bad news, with improvement in the specific areas of information giving and responding to emotional cues. Although cumulative scores for discussions about patient preferences for treatment did not increase, residents demonstrated enhanced specific skills including discussing probability, presenting clinical scenarios, and asking about prior experience with end-of-life decision making. | N/A | N/A |
| 2 | Allenbaugh, J., et al. (2019) Health Literacy and Clear Bedside Communication: A Curricular Intervention for Internal Medicine Physicians and Medicine Nurses. | Mixed methods study | Reports issued by the American Medical Association and the Institute of Medicine call for greater efforts to educate health care professionals about low levels of health literacy and to provide evidence-based techniques for effective communication with the many patients in this category. In an effort to improve bedside communication and the overall patient experience, we designed and implemented a curriculum for medicine inpatient attendings, internal medicine resident physicians, and bedside medicine nurses focusing on teaching clear communication skills to be used as a universal precaution for optimizing patients’ understanding of their medical care. | The intervention consisted of a beside communication curriculum among 37 attending medicine physicians, 76 internal medicine residents, and 85 bedside nurses. The 1- to 1.5-hour curriculum included a didactic session to teach health literacy principles, video demonstrations, group discussion, and role-play. Attending physicians’ health literacy knowledge, attitudes, and confidence were evaluated using pre- and postsurveys. Evaluation of the curriculum included Likert-type questions and free-text responses. | Medicine inpatient attendings, internal medicine resident physicians, and bedside medicine nurses | A brief, low-cost curricular intervention focusing on clear communication skills and health literacy principles resulted in significant improvements in knowledge and attitudes of attending physicians and was readily incorporated by resident physicians and nurses. This curriculum can be easily implemented in a variety of settings to improve bedside patient-physician communication. | 15 | 24 |
| 3 | Ammentorp, J., et al. (2007) The effect of training in communication skills on medical doctors' and nurses' self-efficacy. A randomized controlled trial. | Randomised controlled trial (quantitative) | Several studies have evaluated the effect of communication skills training for health professionals, and a few of these were randomized studies measuring the effect of the courses on patient outcomes. However, lack of evidence of the effects of such interventions on patient health care behaviour and health status has given rise to the assumption that the clinicians’ perception of own skills (in terms of self-efficacy) can reduce the overall effectiveness of a training programme and thereby explain the lack of a direct relationship between communication skills training and patient outcome. The aim of this study was to investigate the effect of communication skills training on doctors’ and nurses’ self-efficacy, to explore how training courses influence the initial experience of self-efficacy and to identify determinants of health professionals’ self-efficacy. | The study was conducted as a randomized trial. Clinicians in the intervention group received a 5 day communication course and the control group received no intervention. The impact of the intervention was evaluated by means of questionnaires measuring the effect of communication courses on changes in doctors’ and nurses’ self-efficacy. | Paediatric doctors and nurses | Communication skills training can improve clinicians’ evaluation of his or her ability to perform a specific communication task—measured as self-efficacy. | 17 | N/A |
| 4 | Arnold, R. M., et al. (2015) The Critical Care Communication project: improving fellows' communication skills. | Quantitative study | Most educational interventions for intensive care professionals have relied on didactic lectures or role modeling for passive learners, although neither of these strategies have been shown to improve communication skills. Evidence-based Communication skills interventions are urgently needed. The aim of this study was to develop an evidence-based communication skills training workshop to improve the communication skills of critical care fellows. | Pulmonary and critical care fellows (N = 38) participated in a 3-day communication skills workshop between 2008 and 2010 involving brief didactic talks, faculty demonstration of skills, and faculty- supervised small group skills practice sessions with simulated families. Skills included the following: giving bad news, achieving consensus on goals of therapy, and discussing the limitations of life-sustaining treatment. Participants rated their skill levels in a pre-post survey in 11 core communication tasks using a 5-point Likert scale. | Pulmonary and critical care fellows | Of 38 fellows, 36 (95%) completed all 3 days of the workshop. We compared pre and post scores using the Wilcoxon signed rank test. Overall, self-rated skills increased for all 11 tasks. In analyses by participant, 95% reported improvement in at least 1 skill; with improvement in a median of 10 of 11 skills. Ninety-two percent rated the course as either very good/excellent, and 80% recommended that it be mandatory for future fellows. | 12 | N/A |
| 5 | Back, A. L., et al. (2007) Efficacy of communication skills training for giving bad news and discussing transitions to palliative care. | Mixed methods study | Few studies have assessed the efficacy of communication skills training for postgraduate physician trainees at the level of behaviors. The purpose is to evaluate a communication skills training programme called Oncotalk. To evaluate Oncotalk, the authors used a system of content-based coding of audiorecordings of encounters with standardized patients because self-assessment often does not correlate with objective measures. This study reports the primary outcome of this evaluation, learner acquisition of communication skills, for the 2 different tasks of delivering bad news and discussing transitions to palliative care. | The primary outcomes were observable participant communication skills measured during standardized patient encounters before and after the workshop in giving bad news and discussing transitions to palliative care. The standardized patient encounters were audiorecorded and assessed by blinded coders using a validated coding system. Before-after comparisons were made using each participant as his or her own control. | Medical oncology fellows | Compared with preworkshop standardized patient encounters, postworkshop encounters showed that participants acquired a mean of 5.4 bad news skills (P.001) and a mean of 4.4 transitions skills (P.001). Most changes in individual skills were substantial; for example, in the bad news encounter, 16% of participants used the word “cancer” when giving bad news before the workshop, and 54% used it after the workshop (P.001). Also in the bad news encounter, blinded coders were able to identify whether a standardized patient encounter occurred before or after the workshop in 91% of the audiorecordings. | 13.5 | 24 |
| 6 | Back, A. L., et al. (2003) Teaching communication skills to medical oncology fellows. | Qualitative study | The overall goal for the project was to improve oncology fellows’ communication skills with patients who have incurable or progressive cancer. To focus on practical skills that fellows would view as essential, the study identified key communication tasks. | To design the educational intervention, the authors used a conceptual model that incorporates learner knowledge, attitudes, and personal experience as factors influencing learner self-efficacy, which can be refined and enhanced by teaching and practice with feedback to influence communication skills. | Medical oncology fellows | This article has described a successful communication skills program designed for oncology fellows that draws from previously reported research, incorporates the clinical realities of practice in cancer centers, and attends to the needs of oncology fellows at their particular point in professional development. | N/A | 18 |
| 7 | Barbosa, M., et al. (2019) Effectiveness of a brief training program in relational/communication skills for medical residents. | Qualitative study | To assess the effectiveness of a brief training program in relational/communication skills (RCS) for medical residents. | This longitudinal study enrolled 64 medical residents who participated in a RCS training program in small groups. Teaching was based on interviews with standardized patients and reflective practice. Video-recorded consultations were coded according to the Verona-Coding-Definitions-of-Emotional-Sequences (VR-CoDES) and a coding system developed to assess ten communication skills for breaking bad news. The outcome measures were: independent raters’ score in RCS for breaking bad news and the percentage of providing space and empathic responses, by comparing baseline (T1) skills with those after three-days (T2) and three-months (T3). | Medical residents | After the training program residents provided more space for further disclosure of cues and concerns according to VR-CoDES definitions. There were significant improvements in seven of the ten communication skills for breaking bad news. All of these improvements were observed in the long term as well. | N/A | 20 |
| 8 | Bayona, J. and Goodrich, T. J. (2008) The integrative care conference: An innovative model for teaching at the heart of communication in medicine. | Qualitative study | During one-to-one clinical supervision in the last 8 years, the authors noticed that their residents used the screening-out template during the clinical interview as well as during precepting. Therefore, the authors challenged themselves to develop a complementary experiential method to enhance the teaching of patient–physician communication with our residents. They implemented an interdisciplinary team to meet with residents and their patients to help residents obtain information from their patients and connect with them at a level not customary in usual practice. They called this new format the Integrative Care Conference (ICC). | By focusing on the biomedical aspects of a disease, physicians often dismiss the emotional effect that patients have on them and the stories that provide meaning to the patients’ experience with illness. This approach often leads to strained relationships, medical errors, and dehumanized health care. This paper describes the Integrative Care Conference, an interdisciplinary format for teaching enhanced communication between residents and their patients. | Second and third year family medicine residents | Three findings emerged: (a) The gap between what the resident knows about the patient and what is relevant to the patient’s health care is wide. (b) Despite this gap, patients express great appreciation for their physician. (c) After learning about their patients’ life and relationships, residents developed more humanistic approaches to their patient that reshaped treatment. | N/A | 20 |
| 9 | Beaudoin, P. L., et al. (2019) Teaching communication skills to OTL-HNS residents: Multisource feedback and simulated scenarios. | Qualitative study | Effective communication has been linked to a reduction in adverse events and improved patient compliance. Currently in Otolaryngology – Head and Neck Surgery (OTL-HNS) residency programs, there is limited explicit teaching of communication skills. The objective of this study was to implement an educational program on communication skills for residents using multisource assessment in several simulation-based contexts throughout residency. | For three consecutive years, OTL-HNS residents were recruited to participate in a total of nine simulation-based clinical scenarios in which communication skills could be honed. This educational program was designed to provide instruction and practice of challenging ethics scenarios, with communication efficacy as a secondary goal. To facilitate this goal, a multisource assessment was paired with a debriefing process that involved attending staff, observing and participating residents, standardized patients, and invited content experts. | Otolaryngology residents | Implementing an educational program focused on communication skills using a multisource assessment in various contexts has shown to be potentially effective, and resulted for yearly improvement and consolidation of performance of OTL-HNS residents as judged by faculty and residents. The inclusion of a multisource assessment in the simulation curriculum is key to allow for the representation of different perspectives on communication skills, for both the assessment and the debriefing process. Future studies are needed to explore the possibility of fully integrating this educational program into residence training in order to support deliberate communication skills teaching. | N/A | 21 |
| 10 | Berkhof, M., et al. (2011) Effective training strategies for teaching communication skills to physicians: an overview of systematic reviews. | Systematic review | The aim of the present review was to identify from the literature effective approaches for teaching communication skills to qualified physicians. In this review, communication skills training is defined as the entire training programme that physicians attend. | PubMED, PsycINFO, CINAHL, and COCHRANE were searched in October 2008 and in March 2009. Two authors independently selected relevant reviews and assessed their methodological quality with AMSTAR. Summary tables were constructed for data-synthesis, and results were linked to outcome measures. As a result, conclusions about the effectiveness of communication skills training strategies for physicians could be drawn. | Physicians | Training programmes were effective if they lasted for at least one day, were learner-centred, and focused on practising skills. The best training strategies within the programmes included role-play, feedback, and small group discussions. | N/A | N/A |
| 11 | Berlacher, K., et al. (2017) The Impact of Communication Skills Training on Cardiology Fellows' and Attending Physicians' Perceived Comfort with Difficult Conversations. | Qualitative study | Cardiologists need to decide which treatments are appropriate for seriously ill patients and whether they align with patient goals. Reconciling medical options with patients’ wishes requires skilled communication. Although there is evidence that communication is teachable, few cardiologists receive formal training. The aim of this study is to demonstrate that providing communication skills training to cardiologists is feasible and improves their perceived preparedness (PP) for leading difficult conversations. | CardioTalk is a workshop to improve communication through short didactic sessions followed by interactions with standardized patients. Competencies include giving bad news, defining goals of care, responding to emotion, supporting religious beliefs, and withdrawing therapies. | Cardiology attendings and fellows | Eight cardiology attendings and 20 cardiology fellows participated. Eighty-nine percent reported having any prior education in communication. Fellows reported more prior education than attendings (100% vs. 62.5%, p = 0.017). Level of PP improved in all competencies for all learners. Ninety-six percent of respondents could recommend the training to peers. All attendings felt that it should be required for cardiologists in the cardiac intensive care unit and reported improved preparedness to teach communication to learners. | N/A | 17 |
| 12 | Boissy, A., et al. (2016) Communication Skills Training for Physicians Improves Patient Satisfaction. | Mixed methods study | Skilled physician communication is a key component of patient experience. Large-scale studies of exposure to communication skills training and its impact on patient satisfaction have not been conducted. This study aimed to examine the impact of experiential relationship-centered physician communication skills training on patient satisfaction and physician experience. | An 8-h block of interactive didactics, live or video skill demonstrations, and small group and large group skills practice sessions using a relationship centered model. | Attending physicians | System-wide relationship-centered communication skills training improved patient satisfaction scores, improved physician empathy, self-efficacy, and reduced physician burnout. Further research is necessary to examine longer-term sustainability of such interventions. | 14.5 | 17 |
| 13 | Bragard, I., et al. (2006) Teaching communication and stress management skills to junior physicians dealing with cancer patients: a Belgian Interuniversity Curriculum. | Descriptive study | Concerning the impact of communication skills training programs on physicians’ level of stress and burnout, results are inconsistent. If stress and burnout among physicians have to be prevented, increased resources will have to be required to develop training not only in communication skills but also in stress management skills. No training program integrating both communication and stress management skills has been designed yet. Therefore, a specific training bringing together a stress management skills and a communication skills training course has been designed for junior physicians specializing in various disciplines: a Belgian Interuniversity Curriculum (BIC). | The aim of the stress management skills training course is to promote better management of stressful situations and difficult interviews with cancer patients and their relatives by choosing the more adapted coping strategy. The communication skills training course aims at improving knowledge related to psychosocial consequences of cancer and effective communication skills and at developing facilitative communication behaviors with patients and their relatives. The whole program is designed to maintain the newly acquired skills and to promote the transfer of these skills to clinical practice. Finally, the BIC aims to increase physicians’ work-related quality of life and to enhance patients’ satisfaction with care. | Junior physicians involved in cancer care | To summarize, few physicians have received formal training in communication during their curriculum. This might in part explain the substantial communication problems between physicians and patients contributing to an insufficient detection of psychological disturbances, patient dissatisfaction with care, poor compliance, and increased risks of litigation for malpractice. This insufficient training in communication skills also contributes to everyday stress, lack of job satisfaction, and burnout among physicians, particularly when they have to manage breaking bad news. | N/A | N/A |
| 14 | Brown, R., et al. (2010) Discussing prognosis in an oncology setting: Initial evaluation of a communication skills training module. | Quantitative study | Although cancer patients from Western countries have high needs for prognostic information, the frequency of such discussions in oncology consultations is variable. Prognosis is a difficult discussion area for both physicians and patients. The primary aim of this research is to describe the development and contents of a CST module designed to assist health-care professionals with discussing prognosis. The second aim of the paper is to present pilot self-report data regarding changes in participants’ confidence in discussing prognosis and their intention to use new skills learned as a results of training. | One hundred and forty-two clinicians from Memorial Sloan-Kettering Cancer Center and the New York City area voluntarily participated in the training module over a twoyear period. The module was based on current literature and followed the Comskil model previously utilized for other doctor–patient CSTs. Participants completed pre and post surveys to evaluate their own confidence as well as the helpfulness of the module. | Multi-speciality clinicians working in the oncology setting | Based on a retrospective pre–post measure, participants reported an increase in their confidence about discussing prognosis (t14159.331, po0.001). At least 92% of participants reported their satisfaction with components of the module by either agreeing or strongly agreeing with 5/6 different statements included in their evaluation forms. | 12.5 | N/A |
| 15 | Brown, R. F. and Bylund, C. L. (2008) Communication skills training: describing a new conceptual model. | Descriptive study | The purpose of this article is thus to present a new model of CST, the Comskil Model, that the authors developed at Memorial Sloan-Kettering Cancer Center (MSKCC) between 2005 and the present. This model addresses the weaknesses we have identified in the previous literature, in that it provides an overarching framework for organizing communication skills, clearly defines a communication skill, and gives explicit descriptions of communication skills that are common across contexts. In doing so, this model provides the basis for curricula in which teaching and assessing specific skills are aligned. Before implementation by the Comskil laboratory to train MSKCC attendings and fellows, the model was reviewed avorably by three prominent international leaders in CST.  There is a mounting body of research evidence describing the utility of workshop-based training to improve doctors’ communication skills. The Comskil Model adds to this literature by providing a framework supported by interpersonal communication theory. | To make teaching communication skills more explicit, and also to aid in the evaluation of skills uptake, the authors conceive consultation communication as having five components: goals, strategies, skills, process tasks, and cognitive appraisals. | Attending physicians and fellows | The model is effective in achieving these goals by providing clear definitions of core communication components and explaining how these components are integrated to achieve communication goals. | N/A | N/A |
| 16 | Bylund, C. L., et al. (2010) The implementation and assessment of a comprehensive communication skills training curriculum for oncologists. | Qualitative study | The objective of this paper is to report the implementation and assessment of the Comskil Training Curriculum at Memorial Sloan-Kettering Cancer Center. | Twenty-eight attending physicians and surgeons participated in communication skills training modules as part of a train-the-trainer program. Doctors were video recorded inclinical consultations with patients two times before training and two times after training, resulting in 112 video recordings for analysis. Recordings were coded using the Comskil Coding System. | Medical oncologists, surgeons and other specialities | Communication skills related to two of the six major skill sets, Establishing the Consultation Framework and Checking, increased following training. Limited changes emerged in three skill sets, while one skill set, Shared Decision Making, did not change. Doctors who attended more training modules had higher levels of change. Female participants demonstrated three skills more frequently than males post-training. | N/A | 22 |
| 17 | Cameron, N. and McMillan, R. (2006) Enhancing communication skills by peer review of consultation videos. | Qualitative study | The need to develop communication skills teaching has long been recognised by providers of medical education. Communication skills training is associated with enhanced clinical eﬀectiveness. Feedback on video-recordings of consultations has been demonstrated to be an eﬀective means of learning and changing behaviour | Eleven doctors took part in a course that used facilitated small groups to review communication skills and to analyse video-recordings of participant’s consultations. The participants were representative of the majority of the general practice population in that they were not part of the vocational training establishment and therefore had limited opportunities to access this type of educational activity. | Physicians | The results demonstrate that following this intervention all participants reported changes in their consulting behaviour and also submitted a video-recording for external peer review. This study indicates that small group video analysis and external peer review are effective methods of learning, developing and reviewing communication skills and describes a method that is both practical and feasible. This approach not only satisfies the requirements of the General Medical Council and appraisal, but participation in this type of communication skills training can also increase job satisfaction and enhance morale. | N/A | 10 |
| 18 | Cannone, D., et al. (2019) Delivering Challenging News: An Illness-Trajectory Communication Curriculum for Multispecialty Oncology Residents and Fellows. | Qualitative study | Courses, modules, and training programs centered on communication, difficult conversations, and breaking bad news exist for oncology fellows, pediatric oncology fellows, and palliative care fellows. However, few programs report a multispecialty learning and practice environment, more specifically one combining adult oncology, pediatric oncology, radiation oncology, and palliative care fellows. In addition, support from multispecialty faculty crossing all subspecialties of oncology is not reported in other course modules.Importantly, current curricula teach difficult communication scenarios (such as discussing relapse and transition to supportive care) as discrete, unconnected tasks. Thus, such curricula do not replicate the complicated evolving longitudinal doctor-patient relationship associated with the illness continuum. For example, the emotional impact on a learner from discussing relapse of disease in a patient with whom the learner has simulated an ongoing relationship is greater, and the tailoring of wording communication more context based, than discussing relapse in an isolated scripted scenario with an SP. | An eight-module course on communication in oncology practice was delivered over 2 months for palliative and oncology fellows and radiation oncology residents. Learners were given a precourse survey in which they were asked to rate their proficiency in various communication tasks. Each learner then participated in a videotaped precourse objective structured clinical exam (OSCE) on breaking bad news with standardized patients (SPs). The course took place over 8 weeks with weekly didactics and role-play. At the end of the course, a second OSCE took place. After the course was completed, the fellows again filled out a proficiency survey. | Multispeciality oncology residents and fellows | Twenty-two learners participated over 2 years of this course. Participants reported a significant increase in perceived competence in all areas on the postcourse survey. SP feedback on OSCEs pre- and postcourse indicated improvement in skills for learners. Pre- and postcourse OSCE video assessment revealed a significant improvement in global communication skills. | N/A | 17 |
| 19 | Chandawarkar, R. Y., et al. (2011) Pretraining and posttraining assessment of residents' performance in the fourth accreditation council for graduate medical education competency: patient communication skills. | Qualitative study | The goal of this project was to teach surgical residents to incorporate patient centered communication skills into their practice, providing emotional support, transition, and continuity of care, as well as information and education, involving family and friends and respecting patient values and preferences. | Residents initially completed a written baseline survey to assess general communication skills awareness. In step 1 of the study, residents were randomized to 1 of 2 simulations using standardized patient instructors to mimic patients receiving a diagnosis of either breast or rectal cancer. The standardized patient instructors scored residents’ communication skills using a case-specific content checklist and Master Interview Rating Scale. In step 2 of the study, residents attended a 3-part interactive pro- gram that comprised (1) principles of patient communication; (2) experiences of a surgeon (role as physician, patient, and patient’s spouse); and (3) role-playing (3-resident groups played patient, physician, and observer roles and rated their own performance). In step 3, residents were retested as in step 1, using a crossover case design. Scores were analyzed using Wilcoxon signed rank test with a Bonferroni correction. | General surgery residents | Case-specific performance improved significantly, from a pretest content checklist median score of 8.5 (65%) to a posttest median of 11.0 (84%) (P= .005 by Wil- coxon signed rank test for paired ordinal data)(n = 44). Median Master Interview Rating Scale scores changed from 58.0 before testing (P=.10) to 61.5 after testing (P=.94). Difference between overall rectal cancer scores and breast cancer scores also were not significant. Patient communication skills need to be taught as part of residency training. With limited training, case-specific skills (herein, involving patients with cancer) are likely to improve more than general communication skills. | N/A | 17 |
| 20 | Christie, D. and Glew, S. (2017) A clinical review of communication training for haematologists and haemato-oncologists: a case of art versus science. | Systematic review | Communication skills are highly relevant for haematologists and are associated with increased physician and patient satisfaction, positive psychosocial outcomes and possible health outcomes. However, communication skills are reported to remain a challenge for haematologists. This paper firstly reviews communication skill training interventions reported in the literature and, secondly, the currently available communication skill training packages for medical professionals. It also discusses a move towards patient-centred communication approaches, which is highly relevant in haematology. | A search of the literature was conducted on electronic medical and psychological databases: Google Scholar, PubMed, Medline and PsychINFO, A combination of terms was used for the search, which included “haemato*”, “hemato*”, “haemato-oncology”, “hemato-oncology”, “communication skills” and “training”. It was a dilemma for the authors whether to include oncology- and cancer-related training, as a large amount of research in these areas was found. Given the differences in training pathways in the UK, it was decided that interventions would be included only if specifically catering for, or conducted with, haematologists or haemato-oncologists. The inclusion of haemato-oncologists led to the inclusion of interventions conducted with haematology/oncology fellows in the US. | Hematology and hemato-oncology | Five interventional studies were identified that used communication skill training programmes catering for, or conducted with, haematologists or haemato-oncologists. All the studies in this review reported that communication skills improved following the intervention. This was mostly assessed by measuring delivery fidelity, rating the physicians’ use of specific components of the taught skills during real or simulated consultations, or physician confidence in their skills. Only one of the studies investigated any form of patient outcome, reporting improved patient satisfaction and post-consultation anxiety following the training | N/A | N/A |
| 21 | Cinar, O., et al. (2012) Communication skills training for emergency medicine residents. | Qualitative study | Emergency departments (EDs) have unique dynamics with regard to doctor–patient communication. Emergency physicians typically have to obtain information from overstressed patients who they meet for the first time, make fast decisions regarding diagnosis and treatment, give information to patients and relatives, and communicate with the consulting doctors and other medical staff. They must also manage stressful situations that are routine in EDs. They are also expected to establish an appropriate line of communication with special groups of patients, for example, patients with altered consciousness and those who have attempted to commit suicide or are psychotic. Therefore, effective communication is a critical skill for successful management in EDs.  The coverage of communication skills in the US and European programs indicates that effective communication is a prerequisite for emergency medicine specialists. Despite its inclusion in core education programs, there are no methods on how to implement this training program or assess the achievements of participants. | Twenty emergency medicine residents attended a 6-week psychoeducation program that was intended to improve their communication skills. The first three sessions of the psychoeducation program consisted of theoretical education on empathy and communication. Other sessions covered awareness, active communication, and empathic skills on a cognitive behavioral basis using discussion, role play, and homework within an interactive group. The effects of the program were assessed using a communication skills scale, empathy scale, and patient satisfaction survey and were reflected by the reduction in the number of undesirable events between doctors and patients in the emergency department. | Emergency medicine residents | Participation in a communication skills training program was associated with: (1) Improved communication skills of emergency medicine residents, (2) Increased patient satisfaction and decreased com- plaints. | N/A | 13 |
| 22 | Clayton, J. M., et al. (2012) Intensive communication skills teaching for specialist training in palliative medicine: Development and evaluation of an experiential workshop. | Mixed methods study | The Australasian Chapter of the Palliative Medicine (AChPM) Curriculum Development Group identified communication as a core skill that trainees in palliative medicine need to acquire, and proposed the development of a communication skills workshop that should become a compulsory part of training to achieve accreditation as a palliative medicine specialist in Australia and New Zealand. This paper describes the development and subsequent evaluation of this module. | A three-day communication workshop was developed in collaboration with expert communication skills facilitators from the United States and Australia. The teaching consists of: (1) brief plenary presentations providing an evidence-based framework for communication and a demonstration of suggested strategies; (2) small group experiential learning providing opportunities to practice communication skills with clinically relevant simulated patients, self-appraisal, constructive feedback, and reflective exercises; and (3) accompanying course-specific written material. Participants completed de-identified questionnaires before, after, and three months following completion of the workshop. | Trainees and fellows of the AChPM, medical practitioners who were completing a six-month AChPM diploma course in palliative medicine (completion of this diploma does not give palliative medicine specialist status), if space was available, to trainees in related disciplines, such as radiation oncology, medical oncology, and respiratory medicine | Forty-one participants completed the training in two workshops held in 2008 and 2009. Participants said in their questionnaire responses that the training was useful, would be helpful for their communication with patients, and that they would recommend the training to others. Qualitative feedback was highly positive. Selfassessed confidence in communication skills significantly increased following the workshop ( p < .001) and was sustained at three months ( p < .001). | 11.5 | 18 |
| 23 | Clayton, J. M., et al. (2013) Evaluation of a novel individualised communication-skills training intervention to improve doctors' confidence and skills in end-of-life communication. | Mixed methods prospective study | Despite the well-established benefits of effective EOL communication, one- third of patients and their surrogates report dissatisfaction with communication in the ICU, and one-half of patients and their surrogates report not understanding their diagnosis or prognosis. Internal medicine residency training programs typically emphasize biomedical learning, but relatively few provide opportunities for residents to improve outpatient interviewing skills or to address challenging patient encounters. | In this mixed-methods prospective study, second- and third-year medical residents were randomized to participate in a simulation-based communication training or a didactic session. Residents completed a pre–post survey after the sessions evaluating the sessions and reflecting on their use of the word “dying” in family meetings. | Medical residents | A 30-minute educational intervention improves internal medicine residents’ self-reported comfort and preparation in talking about death and dying in the ICU. Residents in simulation-based training were more likely to report they learned new skills as compared to the didactic session. Residents report multiple barriers to using the word “dying” EOL conversations. | 15.5 | 22 |
| 24 | Dacre, J., et al. (2004) Communication skills training in postgraduate medicine: The development of a new course. | Qualitative study | The course was designed to improve participants’ skills in the basic communication techniques required for consultations with patient and relatives, as appropriate to the training grade of the participants (that is, UK senior house officer level). Although the course focused on the PACES examination, the teaching was designed to be extrapolated to other areas within the healthcare arena. | A short course for postgraduate trainees has been designed to address the communication skills requirements of the part 2 clinical examination. The aims, development, and content of the course are described. Emphasis is placed on candidates practising skills with patients and receiving feedback during the course. Evidence suggests that practice with feedback is an essential ingredient of communication skillscourses, and is more effective than other methods such asobserving experts or video examples, or simply discussing issues in communication. | Postgraduate medical physicians preparing for the MRCP examinations | Results of a preliminary evaluation indicate that the course was perceived as valuable by candidates and that the aims, format, and content were appropriate. Although the preliminary evaluation was largely positive, it could be argued that the acid test of the effectiveness of a course is an objective evaluation of skills, observed before and after the course, a development that is being considered for future evaluation of the course. Recommendations for applying this type of training to postgraduate trainees in any branch of medicine are given. | N/A | 18 |
| 25 | Fallowfield, L., et al. (2002) Efficacy of a Cancer Research UK communication skills training model for oncologists: a randomised controlled trial. | Mixed methods study | The communication problems of senior doctors working in cancer medicine are not resolved by time and clinical experience. This study aimed to assess the efficacy of an intensive 3-day training course on communication skills in a randomised controlled trial with a two-by-two factorial design and several outcomes. | 160 oncologists from 34 UK cancer centres were randomly allocated to four groups: written feedback followed by course; course alone; written feedback alone; and control. At each of two assessment periods, consultations with six to ten consecutive, consenting patients per doctor were videotaped. 2407 patients participated. Outcome measures included objective and subjective ratings made by researchers, doctors, and patients. | Oncologists | This trial shows that training courses significantly improve key communication skills. More resources should be allocated to address doctors’ training needs in this vital area. | 17 | 20 |
| 26 | Fallowfield, L., et al. (2003) Enduring impact of communication skills training: results of a 12-month follow-up. | Qualitative study | The need to improve training has been acknowledged worldwide and is reflected in calls from many national and international organisations. | Oncologists (N 1⁄4 160) from 34 cancer centres were allocated to written feedback plus course; course alone; written feedback alone or control. Each clinician had 6 – 10 interviews with patients videotaped at baseline and 3 months postintervention. Analysis of videotapes revealed improvements in the communication skills of clinicians randomised to training (n 1⁄4 80) compared with others (n 1⁄4 80). A 12-month follow-up assessment is reported here. | Oncologists | The overall results show that 12 – 15 months postintervention, clinicians had integrated key communication skills into clinical practice and were applying others. This is the first RCT to show an enduring effect of communication skills training with transfer into the clinic. | N/A | 18 |
| 27 | Fellowes, D., Wilkinson, S. and Moore, P. (2003) Communication skills training for health care professionals working with cancer patients, their families and/or carers | Systematic review | To assess whether communication skills training is effective in changing behaviour of health professionals in cancer care with regard to communication/interaction with patients. | We searched CENTRAL (Cochrane Library Issue 3 2001), MEDLINE (1966 to November 2001), EMBASE (1980 to November 2001), PsycInfo (1887 to November 2001), CINAHL (1982 to November 2001), AMED (1985 ‐ October 2001), Dissertation Abstracts International (1861 to March 2002) and EBM Reviews (1991 to March/April 2001). Reference lists of relevant articles were searched. | Physicians working with cancer patients | | N/A | N/A |
| 28 | Fiorentino, M., et al. (2020) Teaching Residents Communication Skills around Death and Dying in the Trauma Bay. | Quantitative | Delivering difficult news to patients and their families is an essential skill that all physicians must possess. Although holding goals of care conversations with patients and families are part of the core competencies for residents in all specialties, standardized processes, such as use of the Objective Structured Clinical Exam (OSCE) to assess whether the residents have achieved this competency is rare. | This communication curriculum was delivered in three separate phases: (1) didactics using a video education e-module, (2) simulated practice of trauma resuscitation with a high-fidelity mannequin followed by role play of delivering difficult news, (3) an observed skills assessment using standardized patients (SPs). Each phase focused on delivery of difficult news of death and of uncertain/poor prognosis after a resuscitation in the trauma bay. Learners were trauma residents that included postgraduate year (PGY) 1–2 general surgery residents and PGY 1–4 emergency medicine residents at a level 1 trauma center. Outcomes include resident comfort, knowledge, and confidence in delivering difficult news in the trauma setting. | Trauma residents from general surgery and emergency medicine | Thirty-nine trauma residents participated in the three-phase curriculum. There was an increase in the mean scores of resident-reported comfort, knowledge, and confidence in delivering difficult news for the seriously injured. SPs rated 78% of residents as competent to perform delivery of difficult news in the trauma bay independently. | 10 | N/A |
| 29 | Fossli Jensen, B., et al. (2011) Effectiveness of a short course in clinical communication skills for hospital doctors: results of a crossover randomized controlled trial (ISRCTN22153332). | Randomised controlled trial | Evaluations of the interventions have mainly focused on one clinical setting, e.g. delivering bad news or patients with specific problems. Few empirical studies have covered the range of doctor– patient encounters in hospitals, across disciplines, including in the emergency room, bedside on rounds, when performing diagnostic procedures (e.g. echocardiography, electromyography), at dis- charge, or at outpatient clinics. If one generic teaching program could be shown to improve the communication skills of all affiliated doctors, it would simplify the implementation of training programs for hospital doctors. | Crossover randomized controlled trial in a 500-bed hospital with interventions at different time points in the two arms. Assessments were video-based and blinded. Intervention consisted of 20 h of communication training, containing alternating plenary with theory/debriefs and practical group sessions with role-plays tailored to each doctor. | Hospital doctors | Utilizing an outpatient-clinic training model developed in the US, this study demonstrated that a 20-h course could be generalized across medical and national cultures, indicating improvement of communication skills among hospital doctors. | N/A | N/A |
| 30 | Fujimori, M., et al. (2003) Communication Skills Training for Japanese Oncologists on How to Break Bad News. | Qualitative study | Despite significant communication gaps between patients and physicians, there has been little effort to initiate and evaluate teaching programs. Hence, this study investigated whether a communication skills training (CST) could be effectively conducted for Japanese oncologists. However, no studies on the usefulness of CST have ever been reported in Asian countries. | Fifty-eight oncologists participated in the CST. The program was evaluated by measuring participant-rated confidence for communication with patients, burnout, and satisfaction of participants. | Oncologists | Immediately and at 3 months after CST, confidence in communication increased significantly compared with before. The providers’ emotional-exhaustion 3 months after the workshop, however, worsened. The participants’ satisfaction level with the program was high. | N/A | 16 |
| 31 | Fujimori, M., et al. (2014) Development and preliminary evaluation of communication skills training program for oncologists based on patient preferences for communicating bad news. | Qualitative study | The communication skills of physicians delivering bad news about cancer, such as an advanced cancer diagnosis, can affect the degree of a patient’s distress. However, many physicians do not have a standard strategy for delivering bad news to patients and find it difficult to communicate bad news with cancer patients and their relatives. The purposes of this study were to develop a CST workshop program for oncologists to improve patient preferred communication skills when breaking bad news based on the previous studies and to evaluate preliminary feasibility the CST program on the objective performances of physicians and the subjective ratings of their confidence about the communication with patients at the pre- and post- CST. | The CST program was developed, based on the previous surveys on patient preferences (setting up the supporting environment of the interview, making consideration for how to deliver bad news, discussing about additional information, and provision of reassurance and emotional support) and addressing the patient’s emotion with empathic responses, and stressing the oncologists’ emotional support. The program was participants’ centered approach, consisted a didactic lecture, role plays with simulated patients, discussions and an ice-breaking; a total of 2-days. To evaluate feasibility of the newly developed CST program, oncologists who participated it were assessed their communication performances (behaviors and utterances) during simulated consultation at the pre- and post-CST. Participants also rated their confidence communicating with patients at the pre-, post-, and 3-months after CST, burnout at pre and 3 months after CST, and the helpfulness of the program at post-CST. | Oncologists |  | N/A | 21 |
| 32 | Gelfman, L. P., et al. (2014) The effectiveness of the Geritalk communication skills course: a real-time assessment of skill acquisition and deliberate practice. | Quantitative study | Communication skills are critical in Geriatrics and Palliative Medicine because these patients confront complex clinical scenarios. The aim of this study is to evaluate the effectiveness of the Geritalk communication skills course by comparing pre- and post-course real-time assessment of participants leading family meetings. The study also evaluated the participants’ sustained skills practice. | The authors compare participants’ skill acquisition before and after Geritalk using a direct observation Family Meeting Communication Assessment Tool, and assessed their deliberate practice at follow-up. | First year geriatric and palliative medicine fellows | Pre- and post-course family meeting assessments were compared. An average net gain of 6.8 skills represented a greater than 20% improvement in use of applicable skills. At two-month follow-up, most participants reported deliberate practice of fundamental and advanced skills. | 14 | N/A |
| 33 | Ghofranipour, F., et al. (2018) Improving interns' patient-physician communication skills: Application of self-efficacy theory, a pilot study | Qualitative study | Research on the effectiveness of theory-based interventions on physicians and medical students’ self-efficacy in communication skills is very limited. This study is an attempt to fill this gap by assessing the effect of a self-efficacy theory-driven educational intervention on self-efficacy and communication skills among medical interns. This study also assessed patients’ satisfaction with interns’ communication skills as the outcome measure of the intervention. | This study was conducted among 70 medical intern students. Being selected by human judgment and homogenous sampling method, participants were assigned into control and intervention groups. Participants in the invention group received an e-book, two one-on-one training sessions and feedback on their shared experiences in group discussions by peers. The Kirkpatrick model was applied to evaluate the intervention. Participants’ knowledge, self-efficacy and communication skills as well as their patients’ satisfaction were assessed. Data from self and observational assessments were compared in and between groups at different time-points | Medical interns | Participants’ knowledge, self-efficacy and communication skills as well as their patients’ satisfaction were improved significantly in the intervention group, compared to that of the control group. Correlation coefficient between interns’ self-efficacy and communication skills scores was 0.74 (P = 0.03). | N/A | 18 |
| 34 | Ghoneim, N., et al. (2019) Teaching NICU Fellows How to Relay Difficult News Using a Simulation-Based Curriculum: Does Comfort Lead to Competence? | Mixed methods study | Neonatal Intensive Care Unit (NICU) clinicians must frequently relay difficult news to patient families, and the need for formal training for NICU trainees to develop this skill has been established. Although previous studies have shown improved trainee self-efficacy and comfort in handling difficult conversations after formal communication training, it remains unclear whether these interventions lead to improved objectively assessed short-term and long-term performance.  This study aimed to objectively assess via evaluations by expert raters the immediate and long-term effect of a simulation-based communication curriculum for NICU fellows on their performance in communicating difficult news. | A simulation-based intervention emphasizing the SPIKES protocol for delivery of bad news was implemented for 15 fellows in the 3-year Baylor College of Medicine Neonatal-Perinatal Medicine fellowship program in the 2013–2014 academic year. | Neonatal-Perinatal Medicine fellows | The results of this study highlight the importance of objective assessments in evaluating the utility of a simulation-based communication curriculum and the need for longitudinal curricula to promote retention of the concepts and skills being taught. | 12 | 19 |
| 35 | Gulbrandsen, P., et al. (2013) Long-term effect of communication training on the relationship between physicians' self-efficacy and performance | Observational study | Patient-centered communication is an integral aspect of medical care and has been promoted for decades. However, changing the standard of communication has been slow and difficult and the long term impact of communication skills training on physician’s assessment of their own communication skills is unknown. The aims of this study were to explore whether the association between physician communication skills self-efficacy and actual performance changed following a communication skills course, and which variables could predict a change in self-efficacy from baseline to follow-up. This is the likely first study to explore long term consequences to self-efficacy and accuracy of that self-assessment after a long follow-up period. | 62 hospital physicians were exposed to a 20-h communication skills course according to the Four Habits patient-centered approach in a crossover randomized trial. Encounters with real patients before and after the intervention (mean 154 days) were videotaped, for evaluation of performance using the Four Habits Coding Scheme. Participants completed a questionnaire about communication skills selfefficacy before the course, immediately after the course, and at 3 years follow-up. Change in self-efficacy and the correlations between performance and self-efficacy at baseline and follow-up were assessed.  This is an observational study following a crossover randomized controlled trial in 2007–2008 [25] with follow-up data collected in 2011. | Physicians in non-psychiatric departments | | N/A | N/A |
| 36 | Haglund, M. M., et al. (2015) Difficult conversations: a national course for neurosurgery residents in physician-patient communication | Quantitative study | The aim of this study is to describe the design, content, implementation, and evaluation of a national curriculum for teaching practical skills in empathic communication to residents in neurosurgery. | Based on needs assessed through a national survey of neurosurgery program directors, videotaped scenarios using standardized patients illustrating good and bad communication skills were developed. Presurveys and postsurveys were conducted querying paticipants on their level of competence and the specific behaviors they would attempt to change following participation. A subgroup of residents was evaluated before and after the training based on videotaped role-play exercises. | Neurosurgery residents | Most of the neurosurgery program directors responding to the survey indicated that an interactive online communication-training module would be of value (77%). A total of 93 residents participated in communication training as part of the Neurosurgery Boot Camps. Approximately hald of the residents reported having no formal physician-patinet communication training. Presurvey and postsurvey results showed significant improvemnet in several of the communication scenarios. Those who participated in role-play showed significant improvement in "asking open-ended questions," "listening," "fire warning shot," "allowing patient to absorb," and "explaining in clear language". | 10.5 | N/A |
| 37 | Han, P. K. J., et al. (2005) The Palliative Care Clinical Evaluation Exercise (CEX): An experience-based intervention for teaching end-of-life communication skills | Interventional study | The purpose of this study was to pilot test a novel clinical, experience-based educational intervention, the Palliative Care Clinical Evaluation Exercise (CEX). This intervention was designed to allow faculty to precounsel, observe, evaluate, and give feedback to internal medicine residents in their actual discussions with seriously ill patients and their patients’ families. This intervention was the initial phase of a larger project aimed at teaching and reinforcing the importance of palliative care communication skills among internal medicine residents. | The authors collected feasibility measurements at the time of intervention, and interns’ attitudes were measured before and one week after intervention and at the end of the intern year. They piloted the Palliative Care CEX among first-year residents and collected initialdata on its feasibility and perceived educational value in order to explore the merit of future efforts to refine, validate,and implement the intervention more broadly. | First year Internal Medicine residents | Forty-four residents (73%) completed the intervention. Discussions averaged a total of 49.5 minutes (SD 24.1), divided among 12.7 minutes (SD 7.5) for prediscussion counseling between the resident and faculty observer, 25.6 minutes (SD 16.1) for the resident– patient discussion, and 12.1 minutes (SD 5.7) for postdiscussion feedback. Residents rated the Palliative Care CEX favorably (3 on a five-point scale) on ease of arranging the exercise, educational value, quality of the experience, effect on their comfort with discussions, importance to theireducation, and value of preceptor feedback. Self-ratings of communicationcompetence showed improvement one week after the intervention. | N/A | N/A |
| 38 | Hardoff, D. and Schonmann, S. (2001) Training physicians in communication skills with adolescents using teenage actors as simulated patients | Qualitative study | Role-play exercises with simulated patients may serve the purpose of training professionals to develop appropriate communication skills with adolescents. Authentic adolescent responses toward the physicians may be achieved by actors who themselves are in their teenage years. This study describes the authors' experience in continuing medical education programmes for primary care physicians aimed at improving their skills in communicating with adolescents, using simulation methodology with teenage actors. | Eight 16±17-year-old actors from the drama department of a high school for the arts were trained to simulate 20 cases with characteristic adolescent medical problems, as well as con®dentiality issues and home and school problems. The actors performed in front of large groups of 20±30 paediatricians, family practitioners, or gynaecologists in continuing medical education. Diagnostic issues as well as therapeutic and management approaches were discussed, while the actors provided feedback to the trainees about their understanding and their feeling regarding the issues raised during the exercises. | Paediatricians, family practitioners, or gynaecologists | Normally, smaller learning groups are more suitable for such training purposes; nevertheless the participants could appreciate learning the principles of careful listening, a non-judgmental approach and assuring confidentiality. A collaboration of medical schools and postgraduate programmes with high schools which have drama departments may be fruitful in the teaching of adolescent medicine with special emphasis on communication skills with teenagers. | N/A | 15 |
| 39 | Haskard, K. B., et al. (2008) Physician and patient communication training in primary care: effects on participation and satisfaction. | Quantitative study | Several important questions about communication training remain unanswered. Communication training for medical patients has received far less research attention, although teaching patients to participate effectively in the medical visit has been shown to improve their satisfaction, participation, question-asking, adherence, and health outcomes and reduce health care disparities. There is no empirical evidence about whether trained physicians and patients would complement each other or instead show problematic interactions and create “conflict” in the system. Further, outcomes of physician and patient training that go beyond patient satisfaction, such as patients’ perceptions of control and choice, physicians’ satisfaction with the medical visit, and physicians’ professional satisfaction and stress, are essential to examine. Related research indicates that greater patient involvement, for example, can positively affect physician satisfaction.  The aim of this study is to assess the effects of a communication skills training program for physicians and patients. | A randomized experiment to improve physician communication skills was assessed 1 and 6 months after a training intervention; patient training to be active participants was assessed after 1 month. Across three primary medical care settings, 156 physicians treating 2,196 patients were randomly assigned to control group or one of three conditions (physician, patient, or both trained).  Patient satisfaction and perceptions of choice, decision-making, information, and lifestyle counseling; physicians’ satisfaction and stress; and global ratings of the communication process. | Primary care specialties (obstetrics/gynecology, family medicine, internal medicine) | The following significant ( p .05) effects emerged: physician training improved patients’ satisfaction with information and overall care; increased willingness to recommend the physician; increased physicians’ counseling (as reported by patients) about weight loss, exercise, and quitting smoking and alcohol; increased physician satisfaction with physical exam detail; increased independent ratings of physicians’ sensitive, connected communication with their patients, and decreased physician satisfaction with interpersonal aspects of professional life. Patient training improved physicians’ satisfaction with data collection; if only physician or patient was trained, physician stress increased and physician satisfaction decreased. | 17 | N/A |
| 40 | Hoffman, M., et al. (2004) Teaching communication skills: An AACE survey of oncology training programs | Qualitative study | The extent of communication skills training (CST) in American oncology fellowship programs is unknown. This aim of this study is to report here the first survey of the prevalence of CST in the various oncology specialties. | A survey was sent to program directors of medical oncology, radiation oncology, gynecologic oncology, and surgical oncology training programs regarding (1) the presence and method(s) of CST in their programs, (2) their attitude about mandatory CST, and (3) their attitude about a mandatory assessment of communication skills competence as a prerequisite for specialty certification. | Medical oncology, radiation oncology, gynecologic oncology, and surgical oncology | Only a third of programs contained some form of CST. Surgical oncology programs were particularly lacking. Lack of faculty time was cited as the major barrier to implementing CST. A majority of program directors support mandatory CST but not a core competence requirement for certification. In summary, this survey found a lack of or inadequacy in communication skills teaching in many American oncology training programs. This finding underscores a need to develop a core educational curriculum, which should ideally incorporate a reliable, valid method(s) to assess competence. | N/A | 16 |
| 41 | Hope, A. A., et al. (2015) Let's Talk Critical. Development and Evaluation of a Communication Skills Training Program for Critical Care Fellows | Mixed methods study | Although expert communication between intensive care unit clinicians with patients or surrogates improves patient- and family-centered outcomes, fellows in critical care medicine do not feel adequately trained to conduct family meetings. This study aimed to develop, implement, and evaluate a communication skills program that could be easily integrated into a U.S. critical care fellowship. | This study developed four simulation cases that provided communication challenges that critical care fellows commonly face. For each case, the authors developed a list of directly observable tasks that could be heyused by faculty to evaluate fellows during each simulation. Te developed a didactic curriculum of lectures/case discussions on topics related to palliative care, end-of-life care, communication skills, and bioethics; this month-long curriculum began and ended with the fellows leading family meetings in up to two simulated cases with direct observation by faculty who were not blinded to the timing of the simulation. The primary measures of effectiveness were the fellows’ self-reported change in comfort with leading familymeetings after the program was completed and the quality of the communication as measured by the faculty evaluators during the family meeting simulations at the end of the month. | Critical care medicine fellows | Over 3 years, 31 critical care fellows participated in the program, 28 of whom participated in 101 family meeting simulations with direct feedback by faculty facilitators. Our trainees showed high rates of information disclosure during the simulated family meetings. During the simulations done at the end of the month compared with those done at the beginning, our fellows showed significantly improved rates in: (1) verbalizing an agenda for the meeting (64 vs. 41%; Chi-square, 5.27; P = 0.02), (2) summarizing what will be done for the patient (64 vs. 39%; Chisquare, 6.21; P = 0.01), and (3) providing a follow-up plan (60 vs. 37%; Chi-square, 5.2; P = 0.02). More than 95% of our participants (n = 27) reported feeling “slightly” or “much” more comfortable with discussing foregoing life-sustaining treatment and leading family discussions after the month-long curriculum. | 12 | 19 |
| 42 | Hulsman, R. L., et al. (2002) The effectiveness of a computer-assisted instruction programme on communication skills of medical specialists in oncology | Quantitative study | This aim of this paper is to investigate the effect of the CAI training, `Interact-Cancer', on the communication behaviour of medical specialists, and on satisfaction of patients about their physician interaction | Communication behaviour was assessed on 23 observation categories derived from the course content. Consultations of medical specialists with cancer outpatients were videotaped at 4 specific stages, 2 before and 2 after Interact-Cancer, with intervals of 4 weeks. Frequencies were rated as well as judgements about the quality of the performance of each target skill. Satisfaction was measured by the Medical Interview Satisfaction Scale. Data were analyzed by means of multilevel statistical methods. | Oncologists | The behavioural assessment showed course effects on ratings of the physicians' quality of performance. No course effects were found on the frequencies of physicians' behaviours and on the patient satisfaction ratings. | 12.5 | N/A |
| 43 | Ju, M., et al. (2014) Assessing interpersonal and communication skills in radiation oncology residents: a pilot standardized patient program | Quantitative study | There is a lack of data for the structured development and evaluation of communication skills in radiation oncology residency training programs. Effective communication skills are increasingly emphasized by the Accreditation Council for Graduate Medical Education and are critical for a successful clinical practice. This study presents the design of a novel, pilot standardized patient (SP) program and the evaluation of communication skills among radiation oncology residents. | Two case scenarios were developed to challenge residents in the delivery of “bad news” to patients: one scenario regarding treatment failure and the other regarding change in treatment plan. Eleven radiation oncology residents paired with 6 faculty participated in this pilot program. Each encounter was scored by the SPs, observing faculty, and residents themselves based on the Kalamazoo guidelines. | Radiation oncology residents | The program was well received by residents and faculty and regarded as a valuable educational experience that could be used as an annual feedback tool. Poor inter rater agreement suggests a need for residents and faculty physicians to better calibrate their evaluations to true patient perceptions. High scores from faculty members substantiate the concern that resident evaluations are generally positive and nondiscriminating. Faculty should be encouraged to provide honest and critical feedback to hone residents’ interpersonal skills. | 11.5 | N/A |
| 44 | Ju, M., Berman, A. T. and Vapiwala, N. (2015) Standardized Patient Training Programs: an Efficient Solution to the Call for Quality Improvement in Oncologist Communication Skills | Meta analysis | There are few reports on physician communication skills training (CST) programs in oncologic specialties and even fewer on innovative approaches to structuring these training programs. Given the barriers to implementing CSTs, this study discusses how standardized patients (SPs) can be used as efficientand strategic starting points for new CST programs and as expansion opportunities for existing ones. | | Oncologists | SP programs are proven, well-structured starting points for new CST programs, and represent expansion opportunities for existing CST initiatives that, through role-play, can seamlessly and methodically incorporate ongoing provider education and evaluation. | N/A | N/A |
| 45 | Kelley, A. S., et al. (2012) Geritalk: communication skills training for geriatric and palliative medicine fellows | Quantitative study | The educational principles and format of an evidence-based, interactive teaching method was drawn upon to develop a communication skills training program customized for the specific needs of geriatric and palliative medicine fellows. The overall goal of the program, called Geritalk, was to improve fellows’ communication skills with patients with serious illness and their families. This article describes the design, content, and evaluation of the Geritalk communication skills program. | The 2-day retreat, held away from the hospital environment, included large-group overview presentations, small-group communication skills practice, and development of future skills practice commitment. Faculty received in-depth training in small-group facilitation techniques before the course. | Geriatric and palliative medicine fellows | Overall satisfaction with the course was very high (mean 4.8 on a 5-point scale). After the course, fellows reported an increase in self-assessed preparedness for specific communication challenges (mean increase 1.4 on 5-point scale, P < .001). Two months after the course, fellows reported a high level of sustained skills practice (mean 4.3 on 5-point scale). In sum, the intensive communication skills program, customized for the specific needs of geriatric and palliative medicine fellows, improved fellows’ self-assessed preparedness for challenging communication tasks and provided a model for ongoing deliberate practice of communication skills | 9.5 | N/A |
| 46 | Kissane, D. W., et al. (2012) Communication skills training for oncology professionals | Literature review | The purpose of this study was to provide a state-of-the-art review of communication skills training (CST) that will guide the establishment of a universal curriculum for fellows of all cancer specialties undertaking training as oncology professionals today. | Extensive literature review including meta-analyses of trials, conceptual models, techniques, and potential curricula provides evidence for the development of an appropriate curriculum and CST approach. Examples from the Memorial Sloan-Kettering Cancer Center CST program are incorporated. | Fellows of all cancer specialties | A core curriculum embraces CST modules in breaking bad news and discussing unanticipated adverse events, discussing prognosis, reaching a shared treatment decision, responding to difficult emotions, coping with survivorship, running a family meeting, and transitioning to palliative care and end of life. Achievable outcomes are growth in clinician’s self-efficacy, uptake of new communication strategies and skills, and transfer of these strategies and skills into the clinic. Outcomes impacting patient satisfaction, improved adaptation, and enhanced quality of life are still lacking. | N/A | N/A |
| 47 | Kramer, A. W., et al. (2004) Acquisition of communication skills in postgraduate training for general practice | Quantitative study | The evidence suggests that a longitudinal training of communication skills embedded in a rich clinical context is most effective. This study evaluated the acquisition of communication skills under such conditions. | In a longitudinal design the communication skills of a randomly selected sample of 25 trainees of a three-year postgraduate training programme for general practice were assessed at the start and at the end of training. Eight videotaped real life consultations were rated per measurement and per trainee, using the MAAS-Global scoring list. The results were compared with each other andwith those of a reference group of 94 experienced GPs. | General practice trainees | The results of this study indicate that communication skills do not improve in a three-year postgraduate training comprising both a rich clinical context and a longitudinal training of communication skills, and that an unsatisfactory level still exists at the end of training. Moreover, GPs do not acquire communication skills during independent practice as they perform comparably to the trainees. Further research into the measurement of communication skills, the teaching procedures, the role of the GP-trainer as a model and the influence of rotations through hospitals and the like, is required. | 14 | N/A |
| 48 | Lau, F. L. (2000) Can communication skills workshops for emergency department doctors improve patient satisfaction? | Quantitative study | The aim of this study is to assess whether the attending of the communication skills workshops by the emergency department doctors improves patient satisfaction and reduces the number of complaints on doctors’ attitude. | Standard performas were sent to all emergency departments (EDs) in Hong Kong soliciting their numbers of written complaints on doctors’ attitude or communication problems during the nine months before and after a series of communication skills workshops. Patient satisfaction surveys in four representative EDs, before and after the workshops, were collected and the satisfaction rates of doctors’ attitude, explanation and advice were subsequently compared. | Emergency medicine doctors | Communication skills workshops in Hong Kong can improve ED doctors communication skills with a corresponding increase in patient satisfaction and reduction of complaints against ED doctors. | 14 | N/A |
| 49 | Lenzi, R., et al. (2011) Communication training in oncology: results of intensive communication workshops for Italian oncologists | Quantitative study | The aims of this study were to examine the feasibility and efficacy of communication courses in positively affecting oncologists’ attitudes, self-efficacy and knowledge in communication. | Five 3-day intensive communication courses were held for oncologists from different geographical regions in Italy. The courses included formal lectures, small group work, role play and interviews with simulated patients. Participants completed questionnaires before and after the 3-day workshop. | Oncologists | An improvement in self-efficacy, knowledge of communication skills, favourable changes in attitudes towards disclosure of medical information and assessing patients’ concerns and fears were demonstrated at the end of the course. The course was feasible and succeeded in improving parameters associated with effective communication behaviours. | 10.5 | N/A |
| 50 | Levinson, W., Lesser, C. S. and Epstein, R. M. (2010) Developing physician communication skills for patient-centered care | Literature review | Growing enthusiasm about patient-centered medical homes, fueled by the Patient Protection and Affordable Care Act’s emphasis on improved primary care, has intensified interest in how to deliver patient-centered care. Essential to the delivery of such care are patient-centered communication skills. These skills have a positive impact on patient satisfaction, treatment adherence, and self-management. They can be effectively taught at all levels of medical education and to practicing physicians. Yet most physicians receive limited training in communication skills. |  | Physicians | This articles describes what is known and how patient-centered communication skills can be nourished, and reviewed their impact on patient outcomes. It demonstrates that patient-centered communication is more than simply being courteous and honest with one’s patients; it is, in fact, a sophisticated process. It is shown that communication skills can be taught and enhanced during the training of new physicians and through continuing medical education for practicing physicians, and proficiency in these skills can be effectively assessed with the right tools. The development of patient-centered communication skills, like other requirements of patient-centered care, can and should be fostered through a variety of interventions by a broad range of stakeholders: medical educators, medical organizations, health care institutions, regulators, government agencies, and insurers. | N/A | N/A |
| 51 | Liénard, A., et al. (2010) Is it possible to improve residents breaking bad news skills? A randomised study assessing the efficacy of a communication skills training program | Quantitative study | This study aims to assess the efficacy of a 40-h training programme designed to teach residents the communication skills needed to break the bad news. | Residents were randomly assigned to the training programme or to a waiting list. A simulated patient breaking bad news (BBN) consultation was audiotaped at baseline and after training in the training group and 8 months after baseline in the waiting-list group. Transcripts were analysed by tagging the used communication skills with a content analysis software (LaComm) and by tagging the phases of bad news delivery: pre-delivery, delivery and post-delivery. Training effects were tested with generalised estimating equation (GEE) and multivariate analysis of variance (MANOVA). | Residents working with cancer patients | This study shows the efficacy of training programme designed to improve residents’ BBN skills. The way residents break bad news may thus be improved. | 15.5 | N/A |
| 52 | Lim, E. C., Oh, V. M. and Seet, R. C. (2008) Overcoming preconceptions and perceived barriers to medical communication using a 'dual role-play' training course |  | To address the poor candidate performance in the talking stations at the local PACES courses, the authors introduced a ‘medical communication’ course, in which small groups of physician-candidates are taken through the paces of a talking station. Such a participatory course, incorporating practical experience in medical communication with feedback, has been shown to be more effective than passive methods, which are primarily observational and discursive. | The authors conducted four medical communication skills courses from 2004 to 2006. A questionnaire was administered before and after completion of each course. The authors assessed respondents’ confidence levels before and after the course and sought to identify perceived barriers to effective communication among medical trainees in Singapore. Finally, the authors asked if they found participation in the course and its DRP nature to be useful. | Internal Medicine residents | Twenty-six participants, 20 men, 6 women, of mean age 30.2 years (standard deviation (SD) 2.01) completed the survey. The pre-course confidence levels (rated on a scale of 1–10) of 6.23 (SD 1.18) rose significantly to 7.58 (SD 0.95) on completion of the course (P = 0.001, Wilcoxon signed rank test). All respondents felt that they had benefited from participation in the medical communication skills course. 24 (92.3%) respondents deemed it useful to have role-played both the doctor and standardized patient in the exercise. We identified respondents with language difficulties to have benefited the most from the course (P = 0.031, odds ratio 2.906 (95%CI 0.292–5.519), linear regression analysis). | 9.5 | 8 |
| 53 | Lloyd, G., et al (2000) Communication skills training for emergency department senior house officers--a qualitative study | Qualitative study | This study explores the use of direct observation of SHO-patient consultations with feedback from a senior doctor as a teaching tool in the ED setting. | Common weaknesses were identified through review of feedback charts by three trained observers. Alteration in clinical and learning behaviour, as well as senior house officer and observer perceptions of the teaching were evaluated qualitatively by a combination of semistructured interviews and focus groups. | Emergency department senior hous officers | Several common weaknesses were identified, notably the use of closed questions, and poor negotiation and explanation of treatment plan and follow up. The senior house officers perceived improvement in their clinical practice, welcomed feedback, and subsequently set, though did not complete educational contracts. While comfortable with this style of teaching, the observers felt that it did not make efficient use of teaching time. This study identifies common weaknesses in the consultation skills of ED SHOs and confirms the need for training in this area. Direct observation is effective in changing SHO behaviour to this end though self directed learning is not necessarily stimulated. Video recorded consultations with group feedback may be a more effective teaching tool. | N/A | 12 |
| 54 | Maatouk-Burmann, B., et al. (2016) Improving patient-centered communication: Results of a randomized controlled trial | Randomised controlled trial | Patient-centered communication is a key element for improving the quality of care in terms of therapeutic relationship, patient participation, and treatment process. Postgraduate trainings provide an essential way of promoting patient centeredness on the job where learning opportunities are often limited by time, patient volume, and economic pressure. This study aimed to investigate whether a training-induced improvement of patient-centered communication behavior could be demonstrated in a sample of experienced, postgraduate physicians during hospital routines. In the present study, changes in patient centeredness during clinical routines of postgraduate physicians (internal medicine) after a three-day communication training were assessed | A randomized controlled trial was conducted in a primary care clinic. The intervention consisted of a communication training that aimed to enhance patient centeredness in postgraduate physicians. The training was based on a need assessment and the principles of deliberate practice. Workplace-based assessment of physicians’ communication behavior was obtained using the Roter Interaction Analysis System. | Internal Medicine physicians | Three months after the intervention, trained physicians showed significantly increased patient centeredness (F = 5.36, p = .04; d = 0.42). | N/A | N/A |
| 55 | Makoul, G. (2001) The SEGUE Framework for teaching and assessing communication skills | Literature review | This article examines uses and characteristics of the SEGUE Framework, a research-based checklist of medical communication tasks. | This report details 7 years of experience with the SEGUE Framework, a checklist of medical communication tasks that is designed to faciliate the teaching and assessment of communication skills, as well as research on doctor-patient communication. | Physicians and medical students | The SEGUE Framework has a high degree of acceptability, can be used reliably, has evidence of validity, and is applicable to a variety of contexts. | N/A | N/A |
| 56 | Manning, B., et al. (2006) Teaching health communication in a family medicine residency program: report of a work in progress | Qualitative study | Despite the wealth of evidence suggesting that health communication training should be a high priority beginning in medical school, medical students receive limited education in the principles and practices underlying communication competence. Furthermore, with the exception of patient interviewing skills courses and exposure to the electronic health record during clinical rotations, undergraduate health communication–related training tends to be primarily didactic, despite evidence from educational research suggesting that active learning strategies are most effective at teaching skills and changing attitudes. Unfortunately, the elements of skillful health communication are difficult to define and teach, do not come easily for most of us, and must be practiced and adapted to the needs of specific patients.  This article describes an ongoing effort at our family practice residency program to enhance health communication skills training in 3 content areas: health literacy, cultural competence, and behavior change counseling. | This article describes an ongoing effort at the authors' family practice residency program to enhance health communication skills training in 3 content areas: health literacy, cultural competence, and behavior change counseling. During their experience with this curriculum, the focus has remained on these 3 content areas while the learning objectives and teaching approaches have evolved in response to barriers and challenges faced along the way. The lessons we have learned about bringing clinical relevance to health communication skills training should be useful to both residents and residency programs. | Family medicine residents | The residents felt least competent in the areas under the general rubric of health communication. More specifically, residents expressed concerns about their ability to provide behavior change/motivational counseling, to use negotiation skills in discussing patients’ self-management options or treatment alternatives, and to broach or provide counseling on topics such as domestic violence and sexual practices, particularly in the context of cultural, racial, or ethnic differences. | N/A | 5 |
| 57 | Manze, M. G., et al. (2015) Brief provider communication skills training fails to impact patient hypertension outcomes | Randomised controlled trial | Hypertension remains a prevalent risk factor for cardiovascular disease, and improved medication adherence leads to better blood pressure (BP) control. This study sought to improve medication adherence and hypertension outcomes among patients with uncontrolled BP through communication skills training targeting providers. | We conducted a randomized controlled trial to assess the effects of a communication skills intervention for primary care doctors compared to usual care controls, on the outcomes of BP (systolic, diastolic), patient self-reported medication adherence, and provider counseling, assessed at baseline and post-intervention. We enrolled 379 patients with uncontrolled BP; 203 (54%) with follow-up data comprised our final sample. We performed random effects least squares regression analyses to examine whether the provider training improved outcomes, using clinics as the unit of randomization. | Primary care physicians | In neither unadjusted nor multivariate analyses were significant differences in change detected from baseline to follow-up in provider counseling, medication adherence or BP, for the intervention versus control groups | N/A | N/A |
| 58 | Marchand, L. and Kushner, K. (2004) Death Pronouncements: Using the Teachable Moment in End-of-Life Care Residency Training | Mixed methods study | The purpose of this paper is to describe a teaching program for first-year family practice residents on how to perform death pronouncements and communicate with families at the end of life. | Multiple media and methods are used in the 90-minute workshop for first-year family practice residents including poetry, prose, and narratives on doing death pronouncements by senior residents; reviews and discussion of protocols for death pronouncement, autopsy, and organ donation; and a role-play of a death pronouncement with the opportunity for reflection | First year Family Practice residents | Residents consistently provide high ratings for the overall value of workshop. The death pronouncement workshop serves to prepare residents emotionally to deal with dying patients and provides them the skills to effectively and compassionately communicate with those patients’ families while addressing all six ACGME core competencies. | 9 | 7 |
| 59 | McCallister, J. W., et al. (2015) Communication skills training curriculum for pulmonary and critical care fellows | Quantitative study | The purpose of this pre–post intervention study was to evaluate a communication skills curriculum focused on facilitation of family meetings for first-year PCCM fellows. The authors hypothesized that, upon completion of this curriculum, (1) PCCM fellows would demonstrate observable improvement in facilitating simulated family meetings and (2) PCCM fellows’ confidence and selfassessed competence in communication skills would increase | We evaluated a 12-month communication skills curriculum using a pre–post, quasiexperimental design. Subjects for this study included 11 first-year fellows who participated in the new curriculum (intervention group) and a historical control group of five fellows who had completed no formal communication curriculum. Performance of communication skills and self-confidence in family meetings were assessed for the intervention group before and after the curriculum. The control group was assessed once at the beginning of their second year of fellowship. | First year fellows in pulmonary and critical care medicine | Fellows in the intervention group demonstrated significantly improved communication skills as evaluated by two psychologists using the Family Meeting Behavioral Skills Checklist, with an increase in total observed skills from 51 to 65% (P<0.01; Cohen’s D effect size [es], 1.13). Their performance was also rated significantly higherwhencompared withthehistorical control group,who demonstrated only 49% of observed skills (P<0.01; es, 1.55). Fellows in the intervention group also showed significantly improved selfconfidence scores upon completion of the curriculum, with an increase from 77 to 89% (P<0.01; es, 0.87) upon completion of the curriculum | 14.5 | N/A |
| 60 | Merckaert, I., et al. (2005) Factors that influence physicians' detection of distress in patients with cancer: can a communication skills training program improve physicians' detection? | Quantitative study | The basic training program was designed to increase physicians’ knowledge about symptoms and prevalence of distress in cancer care and to initiate improvements in physicians’ assessment skills. The consolidation workshops were designed to improve physicians’ supportive skills, which are needed to handle patients’ distress and to pursue the assessment of perceived cues of distress to allow detection of distress. Thus, the authors hypothesized that consolidation workshops would be required to reach the level of improvement in physicians’ assessment and supportive skills needed to improve the detection of distress. | First, the authors used a randomized design to assess the impact, on physicians’ ability to detect patients’ distress, of a 1-hour theoretical information course followed by 2 communication skills training programs: a 2.5-day basic training program and the same training program consolidated by 6 3-hour consolidation workshops. Then, they investigated the contextual, patient, and communication variables or factors associated with physicians’ detection of patients’ distress were investigated. After they attended the basic communication skills training program, physicians were assigned randomly to consolidation workshops or to a waiting list. Interviews with a cancer patient were recorded before training, afterconsolidation workshops for the group that attended consolidation workshops, and 5 months after basic training for the group that attended basic training without the consolidation workshops. Patient distress was recorded with the Hospital Anxiety and Depression Scale before the interviews. Physicians rated their patients’ distress on a visual analog scale after the interviews. Physicians’ ability to detect patients’ distress was measured through computing differences between physicians’ ratings of patients’ distress and patients’ self-reported distress. Communication skills were analyzed according to the Cancer Research Campaign Workshop Evaluation Manual. | Specialist physicians working with cancer patients | Fifty-eight physicians were evaluable. Repeated-measures analysis of variance showed no statistically significant changes over time and between groups in physicians’ ability to assess patient distress. Mixed-effects modeling showed that physicians’ detection of patients’ distress was associated negatively with patients’ educational level (P 0.042) and with patients’ self-reported distress (P 0.000). Mixed-effects modeling also showed that physicians’ detection of patient distress was associated positively with physicians breaking bad news (P 0.022) and using assessment skills (P 0.015) and supportive skills (P 0.045). | 16 | N/A |
| 61 | Merckaert, I., Libert, Y. and Razavi, D. (2005) Communication skills training in cancer care: where are we and where are we going? | Systematic review | This review gives an overview of recent developments in the field of communication skills training programs designed for cancer health care professionals. | The Web of Knowledge was searched for empirical papers published between January 2002 and February 2005. Twenty-two papers were included in the review describing 13 different studies. | Physicians working in cancer care | Results of this review confirm the usefulness of learner-centred, skills-focused, and practise-oriented communication skills training programs organised in small groups of a maximum 6 participants and lasting at least 20 hours. Such communication skills training programs may therefore be recommended to health care professionals treating cancer patients and their families. | N/A | N/A |
| 62 | Miller, D. C., et al. (2018) Teaching Residents How to Talk About Death and Dying: A Mixed-Methods Analysis of Barriers and Randomized Educational Intervention | Mixed methods prospective study | Various educational interventions involving simulation, skills workshops, and traditional didactics have been shown to improve communication skills, but there is a lack of data evaluating the most effective way to teach EOL communication. Barriers to improving this significant doctor–patient/family interaction may include time and resource constraints in training programs as well as resident physician discomfort. Physicians and resident trainees report they do not receive adequate training in EOL communication, leading them to feel both uncomfortable and unprepared to have these conversations.  This study sought to determine whether simulation or didactic educational interventions improved resident-reported comfort, preparation, and skill acquisition. This study also sought to identify resident barriers to using the word “dying.” | In this mixed-methods prospective study, second- and third-year medical residents were randomized to participate in a simulation-based communication training or a didactic session. Residents completed a pre–post survey after the sessions evaluating the sessions and reflecting on their use of the word “dying” in family meetings. | Second and third year internal medicine residents rotating through the ICU | A 30-minute educational intervention improves internal medicine residents’ self-reported comfort and preparation in talking about death and dying in the ICU. Residents in simulation-based training were more likely to report they learned new skills as compared to the didactic session. Residents report multiple barriers to using the word “dying” EOL conversations. | 10 | 14 |
| 63 | Miller-Matero, L. R., et al. (2019) Motivating residents to change communication: the role of a brief motivational interviewing didactic | Quantitative study | Research has demonstrated that the use of MI skills provides clinically significant benefits within patient care for a range of issues, including substance use, poor health behaviors (e.g., diet, exercise), or difficulty with treatment adherence. Although many providers see the importance of counseling patients regarding health behavior change and prevention efforts, few provide this care. The purpose of this study was to evaluate the benefits of a brief MI didactic for residents in an academic internal medicine patient-centered medical home. | Thirty-two residents completed a 1-h MI training between October 2016 and June 2017 and completed measures on their knowledge of, confidence using, and utilization of MI skills prior to the training, immediately after the training, and at a 1-month follow-up. | Academic internal medicine residents | The residents’ knowledge of MI skills increased from pre- to post- test and also increased from pre-test to 1-month follow-up. The residents’ confidence in using MI skills increased from pre- to post-test and also increased from pre-test to 1-month follow-up. Finally, the utilization of some of the MI skills increased from pre-test to 1-month follow-up. | 10 | N/A |
| 64 | Mitchell, J. D., et al. (2016) The Impact of a Resident Communication Skills Curriculum on Patients' Experiences of Care | Quantitative study | Communication skills are often not taught explicitly in residency training. Although the impact of lapses in communication is high, the obstacles to teaching communication skills remain significant. There have been efforts to develop formal teaching programs in communication skills, but the need to teach these skills remains largely unaddressed in procedural specialties such as anesthesiology. | This study implemented a simulation and webbased curriculum in communication for anesthesia residents and used a patient survey adapted from the Four Habits Coding Scheme to detect changes in patient feedback on residents’ communication skills after the curricular intervention. The 4HCS was adapted into an ambulatory surgical patient survey for patients to provide feedback on anesthesia residents’ communication skills. After administering the survey for 4 months, the authors designed and implemented a simulation and web-based curricular intervention in early 2013. After the intervention, the authors administered the survey for 3 months and analyzed the survey data. They mailed surveys (one for each resident–patient encounter) 1 to 2 weeks after patient discharge from the hospital. | Anesthesia residents | Overall patient satisfaction with residents’ communication skills improved after the curricular intervention. | 14.5 | N/A |
| 65 | Mjaaland, T. A. and Finset, A. (2009) Communication skills training for general practitioners to promote patient coping: the GRIP approach | Quantitative study | The objective of this paper is to develop, perform and test the effects of a communication skills training program for general practitioners (GPs). The program specifically addresses the patients’ coping and resources despite more or less severe psychological or physical illness. | training model was developed, based on cognitive therapy and solution-focused therapy. The training was given the acronym GRIP after its main content. The study involved a quasi-experimental design in which 266 consultations with 25 GPs were video recorded. Forty hours of communication skills training were given to the intervention group. | General practitioners | This pilot training model may help change the GPs’ communicative pattern with patients in some situations. Communication skills training programmes that emphasize patient attributions and personal resources should be developed further and tested in general practice settings with an aim to promote patient coping. | 10 | N/A |
| 66 | Moore, P. M., et al. (2013) Communication skills training for healthcare professionals working with people who have cancer | Narrative review | People with cancer, their families and carers have a high prevalence of psychological stress which may be minimised by effective communication and support from their attending healthcare professionals (HCPs). Research suggests communication skills do not reliably improve with experience, therefore, considerable effort is dedicated to courses that may improve communication skills for HCPs involved in cancer care. A variety of communication skills training (CST) courses have been proposed and are in practice. The aim of this study is to assess whether CST is effective in improving the communication skills of HCPs involved in cancer care, and in improving patient health status and satisfaction. | The authors searched the following electronic databases: Cochrane Central Register of Controlled Trials (CENTRAL) Issue 2, 2012, MEDLINE, EMBASE, PsycInfo and CINAHL to February 2012. The original search was conducted in November 2001. In addition, the authors handsearched the reference lists of relevant articles and relevant conference proceedings for additional studies. The original review was a narrative review that included randomised controlled trials (RCTs) and controlled before-and-after studies. In this updated version, the authors limited their criteria to RCTs evaluating ’CST’ compared with ’no CST’ or other CST in HCPs working in cancer care. Primary outcomes were changes inHCP communication skills measured in interactions with real and/or simulated patients with cancer, using objective scales. This study excluded studies whose focus was communication skills in encounters related to informed consent for research. | Physicians working with cancer patients | Various CST courses appear to be effective in improving some types of HCP communication skills related to information gathering and supportive skills. The authors were unable to determine whether the effects of CST are sustained over time, whether consolidation sessions are necessary, and which types of CST programs are most likely to work. This study found no evidence to support a beneficial effect of CST on HCP ’burnout’, patients’ mental or physical health, and patient satisfaction. | N/A | N/A |
| 67 | Myerholtz, L. (2014) Assessing Family Medicine Residents' Communication Skills From the Patient's Perspective: Evaluating the Communication Assessment Tool | Quantitative study | The Communication Assessment Tool (CAT), a paper-based patient survey, is 1 method to assess residents’ interpersonal and communication skills. To further enhance the interpretation of the CAT, benchmark data are needed. This paper aims to expand upon initial benchmarking data for the use of the CAT as an evaluation tool in family medicine residency programs. | Data were collected on 120 residents from 7 family medicine residency programs. Following an appointment with a resident, 1703 patients completed the CAT. | Family medicine residents | These benchmarking data allow family medicine residency programs to compare the performance of their residents with other programs. The CAT can be used as an evaluation and a learning tool in family medicine and may be applicable to other specialties. | 10 | N/A |
| 68 | Newcomb, A. B., et al. (2017) Talk the Talk: Implementing a Communication Curriculum for Surgical Residents | Mixed methods study | The Accreditation Council for Graduate Medical Education milestones provide a framework of specific interpersonal and communication skills that surgical trainees should aim to master. However, training and assessment of resident non-technical skills remains challenging. This study aimed to develop and implement a curriculum incorporating interactive learning principles such as group discussion and simulation-based scenarios to formalize instruction in patient-centered communication skills, and to identify best practices when building such a program. | The curriculum is presented in quarterly modules over a 2-year cycle. Using the surgical simulation center for the training, the study focused on proven strategies for interacting with patients and other providers. The authors trained and used former patients as standardised patients (SPs) in communication scenarios. | General surgery residents | This approach using Trauma Survivors Network volunteers as SPs could be reproduced in other institutions with similar programs. Faculty enthusiasm and support is strong, and learner participation is active. Continued focus on patient and family communication skills would enhance patient care for institutions providing such education as well as for institutions where residents continue on in fellowships or begin their surgical practice. | 13.5 | 14 |
| 69 | Nikendei, C., et al. (2011) Outcome of parent-physician communication skills training for pediatric residents | Quantitative study | In the field of pediatrics, medical conversation is, to a large extent, an interactional process involving both the child and the child’s parents. The quality of communication skills has direct implications for patient satisfaction, adherence, and course of disease. Conducting diagnostics and therapy with a sick child calls for a differentiated interactional approach at the parent–physician level. However, studies on communication training programs in the specialist area of pediatrics have mainly focused on patient–physician communication, and have largely neglected aspects of parent–physician communication. the employment of actors as ‘standardized parents’ has so far been described in only a few reports in which standardized parents were used to evaluate communication skills in OSCE assessments and in the context of delivering bad news. This study aims to investigate the effects of a parent–physician communication skills training program on OSCE performance and self-efficacy in a group control design. | Parallel to their daily work in the outpatient department, intervention-group experienced clinicians in practice (n = 14) participated in a communication training with standardized parents. Control-group physicians (n = 14) did not receive any training beyond their daily work. Performance was assessed by independent video ratings of an OSCE. Both groups rated their self-efficacy prior to and following training. | Physicians working in the pediatric outpatient department | Regarding OSCE performance, the intervention group demonstrated superior skills in building relationships with parents (p < .024) and tended to perform better in exploring parents’ problems (p < .081). The communication training program led to significant improvement in self-efficacy with respect to the specific training objectives in the intervention group (p < .046). Conclusion: Even in physicians with considerable experience, structured communication training with standardized parents leads to significant improvement in OSCE performance and self-efficacy. | 15 | N/A |
| 70 | Noordman, J., Verhaak, P. and van Dulmen, S. (2011) Web-enabled video-feedback: a method to reflect on the communication skills of experienced physicians | Quantitative study | The purpose of this paper was to describe our web-enabled video-feedback method designed to reflect on the communication skills of experienced physicians. | Participating physicians (n = 28) received a ‘personal web link’ to two of their video-recorded consultations. After watching the consultations physicians received feedback by telephone or in a faceto- face meeting, structured around an individualized feedback report. This report contained scores on the communication behavior of the physician in comparison with colleagues and their own communication behavior observed in a previous study, as well as patients’ opinions about their physician’s communication behavior. The physicians were asked to reflect on their communication skills and to comment on the usefulness and efficiency of the feedback method. | Primary care physicians | Almost all physicians were satisfied with the feedback method and in particular valued the web-enabled link to the video-recorded consultations and the structured written report. Feedback by telephone or face-to-face feedback was considered equally appropriate. This web-enabled video-feedback method is a useful and structured design to reflect on the communication skills of physicians. | 11.5 | N/A |
| 71 | Nørgaard, B., et al. (2012) Communication skills training increases self-efficacy of health care professionals | Quantitative study | Knowing that communication with patients and colleagues could be a potential source of complaints and conflicts, the head of the Department of Orthopaedic Surgery at the Kolding Hospital in Denmark commissioned a 3-day communication skills training course for all staff members. It was expected that the training would result in increased self-efficacy followed by increased patient-centeredness in communication and more respectful intercollegial communication. The specific goal of the training was to enhance the participants’ communication skills in terms of accuracy, efficiency, and supportiveness by giving them some simple yet useful communication principles that were immediately applicable to their daily work in the department. | The study was designed as an effectiveness study with the training course implemented in a real-world context. The staff members attended a 3-day training course in patient-centered communication and communication with colleagues. The effect of the training was evaluated by means of a questionnaire filled out before, immediately after, and 6 months after the course. | Orthopedic surgery | Of the 181 participants, 177 answered the questionnaire before, 165 immediately after, and 150 six months after the course. The mean score for self-efficacy in communication with patients increased from 6.68 to 7.88 (p < .001) and in communication with colleagues from 6.85 to 7.84 (p < .001) immediately following the training course. The effect was still present 6 months after the course was completed. | 11 | N/A |
| 72 | Orgel, E., McCarter, R. and Jacobs, S. (2010) A failing medical educational model: a self-assessment by physicians at all levels of training of ability and comfort to deliver bad news | Quantitative study | Patient surveys consistently show physician communication remains less than ideal. While previous studies have demonstrated a lack of trainee confidence in delivering bad news, this study explores communication skills at all levels of practice and highlights potential barriers to improvement. | Pediatric residents, fellows, and attendings involved in direct patient care at a major academic center participated in a voluntary questionnaire, consisting of self-assessed scales of comfort level, knowledge level, amount of training, and attitudes towards communication education. We also elicited barriers to learning and teaching as well as significant experiences. | Pediatric residents, fellows and attendings | Independent of level of training, this study reveals a lack of self-assessed preparedness from many responsible for delivering bad news to patients and families. A significant barrier to improvement is the disproportionate level of self-assessed comfort versus knowledge level. Educational models should include both didactics to learn the skills and practice-based learning to refine the techniques. | 8 | N/A |
| 73 | O'Shaughnessy, S. M. (2018) Peer teaching as a means of enhancing communication skills in anaesthesia training: trainee perspectives | Quantitative study | This study examines the use of peer teaching of communication skills in anaesthesia training, as a sustainable and economically viable solution to the absence of quality teaching in this area. | All first-year specialist anaesthesia trainees (SATs) in Ireland are required to attend a mandatory professional development programme (PDP) mandated by the College of Anaesthetists of Ireland (CAI) during their first year of training. This taught course focuses on the areas of communication skills, professionalism, self-management, risk management and the structure of health systems. The teaching was delivered over 5 PDP sessions, 2 in year 1 (18 students per session) and 3 in year 3 (11, 12, 20 students in each session, respectively). Two peer teachers designed and delivered a 4-h communications skills session as part of this course. (specific communication skills not mentioned). The first-year anaesthesia trainees completed a survey at the end of the course, which was adapted from a study by Bulte and utilised a Likert rating scale [2]. | First year specialist anesthesia trainees | Of the 79 respondents (36 in Y1 and 43 in Y2), 99% either agreed or strongly agreed that the peer teachers were successful in their role. Ninety-two percent requested formal peer teaching in other areas of training. The trainees regarded a peer teacher as an appropriate information provider (92%), role model (88%), planner (88%) and facilitator (94%), but less so as an assessor (70%). The most consistently stated strength of peer teaching was the relatability of peer teachers with their lack of experience cited as the main weakness. Eighty percent of participants preferred peer teaching to regular expert teaching. | 9 | N/A |
| 74 | Park, I., et al. (2010) Breaking bad news education for emergency medicine residents: A novel training module using simulation with the SPIKES protocol | Quantitative study | Breaking bad news (BBN) in the emergency department (ED) is a common occurrence. This is especially true for an emergency physician (EP) as there is little time to prepare for the event and likely little or no knowledge of the patients or family background information. At the authors' institution, there is no formal training for EP residents in delivering bad news. The authors felt teaching emergency medicine residents these communication skills should be an important part of their educational curriculum. Hence, this paper describes their experience with a defined educational program designed to educate and improve physician’s confidence and competence in bad news and death notification. | A regularly scheduled 5-h grand rounds conference time frame was dedicated to the education of EM residents about BBN. A multidisciplinary approach was taken to broaden the prospective of the participants. The course included lectures from different specialties, role playing for three short scenarios in different capacities, and hifidelity simulation cases with volatile psychosocial issues and stressors. Participants were asked to fill out a self-efficacy form and evaluation sheets. | Emergency medicine residents | Fourteen emergency residents participated and all thought that this education is necessary. The mean score of usefulness is 4.73 on a Likert Scale from 1 to 5. The simulation part was thought to be the most useful (43%), with role play 14%, and lecture 7%. This study shows that teaching physicians to BBN in a controlled environment is a good use of educational time and an important procedure that EP must learn. | 8 | N/A |
| 75 | Patki, A. and Puscas, L. (2015) A Video-Based Module for Teaching Communication Skills to Otolaryngology Residents | Quantitative study | At the authors' institution, they recently developed a training module for residents to improve communication with patients in various settings. This study reports the initial results from the design and implementation of the training module, along with feedback from initial participants as to the usefulness of the module. | During the normal grand rounds schedule, 2 sessions of 1 hour each on "Doctor/ patient communication: a special course for ENT residents" were held. Each session was attended by 11 postgraduate year 1 through postgraduate year 5 residents in the program. Two sets of videos were shown demonstrating "good" and "bad" versions of 4 clinical conversations that commonly occur during surgical care of patient. Each scenario was presented with the "bad" video being shown first and the "good" video second, with a brief time allowed in between for residents to reflect and write notes on their perception of the videos. All residents were then asked to fill out a survey at the end of each session regarding the usefulness of the videos. | Otolaryngology residents | All 11 residents attended both sessions. Of 22 total survey responses, 21 found that the videos were “realistic and engaging” and were a true representation of commonly encountered clinical scenarios. Residents identified multiple themes and behaviors distinguishing “good” vs “bad” communication with patients and felt they could incorporate these into daily practice. A perceived weakness was the lack of opportunity for “role playing” with a video-based module as opposed to standardized patients. | 9 | N/A |
| 76 | Peterson, E. B., et al. (2016) Development of a Comprehensive Communication Skills Curriculum for Pediatrics Residents | Quantitative study | Communication is a core competency that is not systematically taught in many residency programs. A pediatrics program developed a formal, longitudinal communication curriculum and assessed its impact. | A multidisciplinary working group contributed to the development of the RCSC, guided by an institutional needs assessment, literature review, and the Accreditation Council for Graduate Medical Education core competencies. The result was a cohesive curriculum that incorporates didactic, role play, and real-life experiences over the course of the entire training period. Methods to assess curricular outcomes included self-reporting, surveys, and periodic faculty evaluations of the residents. | Pediatric residents | Curricular components have been highly rated by residents (3.95–3.97 based on a 4-point Likert scale), and residents’ self- reported communication skills demonstrated an improvement over the course of residency in the domains of requesting a consultation, providing effective handoffs, handling conflict, and having difficult conversations (intern median 3.0, graduate median 4.0 based on a 5-point Likert scale, P .002). Faculty evaluations of residents have also demonstrated improvement over time (intern median 3.0, graduate median 4.5 based on a 5-point Likert scale, P , .001). | 11.5 | N/A |
| 77 | Price-Haywood, E. G., Harden-Barrios, J. and Cooper, L. A. (2014) Comparative effectiveness of audit-feedback versus additional physician communication training to improve cancer screening for patients with limited health literacy | Quantitative study | Patients often want to engage in discussions with their primary care physician about cancer risk. However, physicians may alter health education about cancer screening based on patient sociodemographics. | Communication intervention PCPs received skills training that included standardized patient (SP) feedback on counseling behaviors. All PCPs underwent chart audits of patients’ screening status semiannually up to 24 months and received two annual performance feedback reports. PCPs experienced three unannounced SP encounters during which SPs rated PCP communication behaviors. The study examined between group differences in changes in SP ratings and patient knowledge of cancer screening guidelines over 12 months; and changes in patient cancer screening rates over 24 months. | Primary care physicians | There were no group differences in SP ratings of physician communication at baseline. At follow-up, communication intervention PCPs were rated higher in general communication about cancer risks and shared decision making related to colorectal cancer screening compared to PCPs who only received performance feedback. Screening rates increased among patients of PCPs in both groups; however, there were no between-group differences in screening rates except for mammography. The communication intervention did not improve patient cancer screening knowledge. | 15 | N/A |
| 78 | Rao, J. K., et al. (2007) Communication interventions make a difference in conversations between physicians and patients: a systematic review of the evidence | Systematic review | The aim of this study was to synthesize the findings of studies examining interventions to enhance the communication behaviors of physicians and patients during outpatient encounters. | The authors conducted searches of 6 databases between 1966 and 2005 to identify studies for a systematic review and synthesis of the literature. Eligible studies tested a communication intervention; were randomized controlled trials (RCTs); objectively assessed verbal communication behaviors as the primary outcome; and were published in English. Interventions were characterized by type (eg, information, modeling, feedback, practice), delivery strategy, and overall intensity. The authors abstracted information on the effects of the interventions on communication outcomes (eg, interpersonal and information exchanging behaviors). Subsequently, they examined the effectiveness of the interventions in improving the communication behaviors of physicians and patients. | Physicians | The interventions were associated with improved physician and patient communication behaviors. The challenge for future research is to design effective patient and physician interventions that can be integrated into practice. | N/A | N/A |
| 79 | Raper, S. E., et al. (2015) Improving Communication Skills: A Course for Academic Medical Center Surgery Residents and Faculty | Quantitative study | To improve physician/patient communication and familiarize surgeons with contemporary skills for and metrics assessing communication, courses were developed to provide academic general surgery residents and faculty with a toolkit of information, behaviors, and specific techniques. If academic faculty are expected to mentor residents in communication and residents are expected to learn good communication skills, then both should have the necessary education to accomplish such a goal. A course in communication, as developed here, quantitatively confirms the effectiveness of this approach to teaching communication skills as well as identifying areas for improvement. Such a course was part of a plan to increase the percentage of “top box” HCAHPS scores and percentile rankings. | Didactic lectures introduced current concepts of physician-patient communication including information on better patient care, fewer malpractice suits, and the move toward transparency of communication metrics. Next, course participants viewed and critiqued “Surgi-Drama” videos, with actors simulating “before” and “after” physician-patient communication scenarios. Finally, participants were provided with a “toolkit” of techniques for improving physician-patient communication including “2– 3–4”—a semiscripted short communication tool residents and other physicians can use in patient encounters—and a number of other acronymic approaches.  Surveys were distributed to course participants in a attempt to ascertain which ideas were considered valuable. There were 6 questions with responses on a 5-point Likert scale, with anchors from "strongly disagree" (1) to "strongly agree" (5). The individual data sheets were summed, and satisfactory scores were considered to be a 4 or 5. Open-ended questions soliciting information about the strengths and opportunities to improve the courses were also asked. | Academic medical centre surgery residents | Each participant was asked to complete an anonymous evaluation to assess course content satisfaction. Overall, 86% of residents participated (68/79), with a 52% response rate (35/68) for the evaluation tool. Overall, 88% of faculty participated (84/96), with an 84% response rate (71/84). Residents voiced satisfaction with all domains. For faculty, satisfaction was quantitatively confirmed (Likert score 4 or 5) in 4 of 7 domains, with the highest satisfaction in “communication of goals” and “understanding of the HCAHPS metric.” The percentage of “top box” Doctor Communication Hospital Consumer Assessment of Health-care Providers and Systems (HCAHPS) scores and national percentile ranking showed a sustained increase more than 1 and 2 years from the dates of the courses. | 8 | N/A |
| 80 | Raper, S. E., Resnick, A. S. and Morris, J. B. (2014) Simulated disclosure of a medical error by residents: Development of a course in specific communication skills | Quantitative study | This course was attended by senior-level residents (entering the postgraduate 4 clinical year), about to embark on the senior clinical phase of their residency, when their supervisory position might require the use of such disclosure skills. As residents with some autonomy and decision-making responsibility, it is likely they will be involved in caring for patients with medical injuries that are ultimately the responsibility of the attending surgeon. Arguably, all residents should learn truth telling from day 1 of residency, but there is little published literature on how to teach this skill. | Before the development of this course, residents had no education in the skills necessary to disclose medical errors to patients. Residents viewed a Web-based cideo didactic session and associated slide deck adn then were filmed disclosing a wrong site surgery to an SP. The filmed encounter was reviewed by faculty, who then along with the SP scored each encounter (5-pt Likert sclae) over 10 domains of physician-patient communication. The residents received individualised written critique, the numerical analysis of their indivifual scenario, and an opportunity to provide feedback over a number of domains. A mean score of 4.00 or greater was considered satisfactory. Faculty and SP assessments were compared with Student t test. | Surgical residents | Residents demonstrated satisfactory competence in 4 of the 10 domains assessed by the course faculty. There were significant differences in the perceptions of the faculty and SP in 5 domains. The residents found this didactic, simulated experience of value. Qualitative feedback from the residents confirmed the realistic feel of the encounter and other impressions. | 11 | N/A |
| 81 | Razack, S., et al. (2007) Coming of age as communicators: differences in the implementation of common communications skills training in four residency programmes | Mixed methods study | This study describes how programme-specific cultural factors (hidden curricula) that emerged as common content were adapted to different training contexts, and outlines an approach to implementing shared training initiatives between programmes in core competencies such as communication skills. | Communications skills training based upon the Kalamazoo consensus statement of communication skills in the clinical encounter was implemented in 4 residency programmes. Field notes of the CST sessions in each programme were analysed and coded for themes, considering the domains of Context, Input, Process and Product (CIPP methodology). Immediate learning outcomes were quantitatively assessed using retrospective pre ⁄post methodology. | Residents of general surgery, internal medicine, obstetrics and gynaecology, and paediatrics. | Important differences were noted in the implementation of CST in the 4 disciplines. The 2 surgical disciplines showed relatively less reflective language and greater concentration on straight skill acquisition, whereas the 2 medical disciplines concentrated on the residents’ role as teachers of communication skills for buy-in. Thematic similarities between disciplines included similar challenges to being good communicators in practice, as identified by residents (e.g. inadequate time and space), as well as lack of formal training. Quantitative learning outcome data from the educational intervention were significant in all groups (P < 0.05). | 11 | 21 |
| 82 | Riess, H., et al. (2011) Improving empathy and relational skills in otolaryngology residents: a pilot study | Quantitative study | Physician empathy and relational skills are critical factors predicting quality of care, patient safety, patient satisfaction, and decreasing malpractice claims. Studies indicate that physician empathy declines throughout medical training, yet little is published about methods to enhance empathy, especially in surgical residency training. To address this important problem, the first author developed an innovative empathy-relational skills training protocol focusing on the underlying neurobiological mechanisms of empathy and the interpersonal processes that positively affect the patient-doctor relationship. | On the basis of recent mirror neuron research, the first author developed an empathy and relational skills protocol focusing on the neurobiology and physiology of emotions, including videos that portray difficult patient-physician interactions. These videos display real-time physiological responses for both members of the dyad, allowing observers to see the degree to which patient and physician are physiologically concordant or discordant with one another. By integrating biometrics, the training aims at deeper levels of physiological awareness and regulation that promise to be more effective than traditional didactic teaching. This neuroscience approach may be particularly effective for training surgical subspecialties as it augments the concept of “being nice” by demonstrating the specific physiological and ameliorative effects on autonomic nervous system activity produced by empathic skills. At baseline and at study completion, residents completed 5 self-report measures. In addition, patients rated the physicians on the CARE measure at baseline and training completion. | Otolaryngology residents | Results showed that a brief series of 3 empathy training sessions can significantly improve physicians’ knowledge of the neurobiology and physiology of empathy, as well as their self-reported capacity to empathize with patients. A trend toward increased patient satisfaction was observed. | 11.5 | N/A |
| 83 | Roter, D. L., et al. (2004) Use of an innovative video feedback technique to enhance communication skills training | Quantitative study | Despite growing interest in medical communication by certification bodies, significant methodological and logistic challenges are evident in experiential methods of instruction. There were three study objectives in this study: 1) to explore the acceptability of an innovative video feedback programme to residents and faculty; 2) to evaluate a brief teaching intervention comprising the video feedback innovation when linked to a one-hour didactic and role-play teaching session on paediatric residents’ communication with a simulated patient; and 3) to explore the impact of resident gender on communication change. | Pre ⁄ post comparison of residents’ performance in videotaped interviews with simulated patients before and after the teaching intervention. Individually tailored feedback on targeted communication skills was facilitated by embedding the Roter Interaction Analysis System (RIAS) within a software platform that presents a fully coded interview with instant search and review features. | First year pediatric residents | The RIAS embedded CD-ROM provides a flexible structure for individually tailoring feedback of targeted communication skills that is effective in facilitating communication change as part of a very brief teaching intervention. | 11.5 | N/A |
| 84 | Roze des Ordons, A. L., et al. (2017) From Communication Skills to Skillful Communication: A Longitudinal Integrated Curriculum for Critical Care Medicine Fellows | Descriptive study | Communication with patients and families in critical care medicine (CCM) can be complex and challenging. A longitudinal curricular model integrating multiple techniques within classroom and clinical milieus may facilitate skillful communication across diverse settings. | In 2014–2015, the authors developed and implemented a curriculum for CCM fellows at the Cumming School of Medicine, University of Calgary, to promote the longitudinal development of skillful communication. A departmental needs assessment informed curriculum development. Five 4-hour classroom sessions were developed: basic communication principles, family meetings about goals and transitions of care, discussing patient safety incidents, addressing conflict, and offering organ donation. Teaching methods—including instructor-led presentations incorporating a consistent framework for approaching challenging conversations, simulation and clinical practice, and feedback from peers, trained facilitators, family members, and clinicians—supported integration of skills into the clinical setting and longitudinal development of skillful communication. Seven fellows participated during the first year of the curriculum. | Critical care medicine fellows | CCM fellows engaged enthusiastically in the program, commented that the framework provided was helpful, and highly valued the opportunity to practice challenging communication scenarios, learn from observing their peers, and receive immediate feedback. | N/A | N/A |
| 85 | Salib, S., et al. (2015) Developing a Communication Curriculum and Workshop for an Internal Medicine Residency Program | Quantitative study | Effective communication between healthcare providers and their patients is related to better patient adherence, decreases in patient psychological distress, alleviation of symptoms, and better recovery from surgical procedures. Although multiple benefits stem from effective communication between providers and their patients, communication breakdowns persist. Given the crucial role communication plays in health care, it is essential to incorporate more effective communication training programs within medical education. Given the multiple benefits associated with good-quality provider-patient communication and the prominent role that evaluative tools such as HCAHPS play within modern healthcare institutions, this project was designed to assess the impact of a communication workshop offered to IM residents. | The authors conducted a communication workshop for internal medicine residents at the University of Texas. Topics covered included the Acknowledge, Introduce, Duration, Explanation, Thank You framework; managing up; resolving conflicts; error disclosure; new medication and discharge counseling; intercultural communication; understanding Hospital Consumer Assessment of Healthcare Providers and Systems scores; and avoiding burnout. Because it would have been logistically difficult to block whole days for the workshop, the various topics were offered to residents during their regular noon conference hour for several consecutive days. After the workshop, participants completed an anonymous questionnaire regarding their perception of the importance of various aspects of communication in patient care. | Internal Medicine residents | The majority of the participating residents perceived the various communication skills explored during the workshop to be highly important in patient care. Concurrently, however, most residents believed that they had initially overestimated their knowledge about these various communication issues. Some demographic differences in the responses also were noted. | 8.5 | N/A |
| 86 | Saypol, B., et al. (2015) A review of three educational projects using interactive theater to improve physician-patient communication when treating patients with irritable bowel syndrome | Systematic review | Three projects were conducted, all with the common aim to use IT to improve physician-patient communication using a patient centered approach. Efforts were made to obtain qualitative data as to the impact of this activity on the learners. The specific programs of IT were designed to: a) to develop and implement a facilitative style of communication, including active listening skills; b) to increase the expression of empathy toward the patient; c) to clarify and accept the patient’s perspective on each issue and engage in a conversation to reach a common understanding and set of goals; d) to learn to acknowledge and manage one’s own emotions in the clinic; and e) to increase cultural sen-sitivity and competency | Three projects are reported. They were collaborations between Theater Delta, the UNC Center for Functional GI and Motility Disorders, the Rome Foundation, the World Gastroenterology Organization, and the American Gastroenterological Association. 8 forced choice and 6 open ended were collected from each participant using a post-performance evaluation form. | Gastrointestinal physicians, medical students | Data indicates that Interactive Theater stimulates constructive dialogue, analysis, solutions, and intended behavior change with regard to communication skills and adapting to patients from multicultural backgrounds. Interactive Theater directly focuses on communication itself (active listening, empathy, recognizing cultural differences, etc.) and shows promise as an effective way to improve awareness and skills around these issues. | N/A | N/A |
| 87 | Schell, J. O., et al. (2018) NephroTalk: Evaluation of a Palliative Care Communication Curriculum for Nephrology Fellows | Quantitative study | Nephrologists care for a medically complex population that faces difficult decisions around treatment options and end-of-life care. Yet communication training within nephrology fellowship is rare. Prior work suggests that communication training in nephrology can improve perceived preparedness to engage in difficult conversations; however, it is unclear if this training results in improved clinical skills.  The primary aim of this study was to evaluate the efficacy of a three-day curriculum for nephrology fellows (NephroTalk) to improve communication skill acquisition for delivering serious news. The study also measured self-reported preparedness for three additional communication tasks taught, including goals of care and transitions at end of life. | Thirty-three first- and second-year fellows from seven academic nephrology programs participated in NephroTalk from 2015 to 2016. Pretraining and post-training encounters to deliver bad news with standardized patients were audiorecorded and evaluated using a modified communication checklist. Fellow experience and self-reported improvement in communication tasks were measured using a five-point Likert scale. | Nephrology fellows | Skill use increased after training for seven of the nine skills measured (P < 0.01). The average number of skills gained after training was 3.6 1.8 skills. With increased communication proficiency, post-training encounters were significantly shorter than pretraining encounters (P 1⁄4 0.03). Fellows reported improved preparedness to engage in all communication tasks taught in NephroTalk curriculum. | 15.5 | N/A |
| 88 | Schellenberg, K. L., et al. (2014) Breaking bad news in amyotrophic lateral sclerosis: the need for medical education | Quantitative study | The manner in which physicians break bad news is an area of discontent for patients with ALS. The future psychological well-being of patients is profoundly affected by this interaction. Lack of physician training is one barrier to breaking bad news effectively. Since general neurologists are often the first to deliver the diagnosis of ALS, it is important that all residents acquire the necessary skills. Although courses have been developed for teaching neurology trainees communication skills, including the skill of breaking bad news, none has been reported in the context of ALS. The first step in determining appropriate education is to assess the existing skill level of neurology residents in delivering an ALS diagnosis.  The main objective of this study was to explore the need for medical education with respect to residents’ ability to communicate the diagnosis of ALS. Secondary areas of interest pertain to this project ’ s use of self-assessment: do ratings provided by the examiners correlate to residents ’ self-ratings; and how do residents perceive the exercise of self-assessment? | Twenty-two resident physicians were videotaped and rated by wo ALS neurologists as they delivered an ALS diagnosis to a standardized patient (SP) during an objective structured clinical examination (OSCE). Residents self-rated immediately after the OSCE, again after viewing their videotape, and completed a survey regarding the OSCE and delivering difficult diagnoses. | Neurology residents | OSCE performance was suboptimal, particularly for communication skills and empathy. The two examiners ’scores correlated except for the empathy subscore. Residents ’self-assessments did not align with the examiners ’scores either before or after watching their videotape. The survey uncovered residents ’apprehension and dissatisfaction with their training in diagnosis delivery. The results highlight a need for resident education in delivering an ALS diagnosis. The lack of correlation between residents ’and examiners ’scoring requires further study. Evaluation of empathy is particularly challenging. Residents agreed that OSCE participation was worthwhile. | 14 | N/A |
| 89 | Schoenborn, N. L., et al. (2015) Incorporating prognosis in the care of older adults with multimorbidity: description and evaluation of a novel curriculum | Quantitative study | This study describes the development and evaluation of an innovative curriculum that teaches internal medicine residents how to assess, communicate and apply prognosis to inform clinical decisions in the care of older adults with multimorbidity. This addresses a significant gap in the literature as we find no previously described curriculum that teaches about incorporating prognosis in the care of this patient population. While existing curricula teach communication or assessment of prognosis in oncology or palliative care, none addresses the unique challenges of incorporating prognosis in the care of older adults with multimorbidity. | The curriculum includes three small-group sessions and a clinical exercise; it focuses on the assessment, communication, and application of prognosis to inform clinical decisions. The curriculum was implemented with 20 first-year residents at one university-based residency (intervention group). Fifty-two first-year residents from a separate residency affiliated with the same university served as controls. Evaluation included three components. A survey assessed acceptability. A pre/post survey assessed attitude, knowledge, and self-reported skills (Impact survey). Comparison of baseline and follow-up results used paired t-test and McNemar test; comparison of inter-group differences used t-test and Fisher’s exact test. A retrospective, blinded pre/post chart review assessed documentation behavior; abstracted outcomes were analyzed using Fisher’s exact test. | First year Internal Medicine residents | The curriculum was highly rated (4.5 on 5-point scale). Eighteen intervention group residents (90 %) and 29 control group residents (56 %) responded to the Impact survey. At baseline, there were no significant inter-group differences in any of the responses. The intervention group improved significantly in prognosis communication skills (5.2 to 6.6 on 9-point scale, p < 0.001), usage of evidence-based prognostic tools (1/18 to 14/18 responses, p < 0.001), and prognostic accuracy (1/18 to 9/18 responses, p = 0.005). These responses were significantly different from the control group at follow-up. Of 71 charts reviewed in each group, prognosis documentation in the intervention group increased from 1/25 charts (4 %) at baseline to 8/46 charts (17 %) at follow-up (p = 0.15). No prognosis documentation was identified in the control group at either time point. Inter-group difference was significant at follow-up (p = 0.006). | 10.5 | N/A |
| 90 | Shaw, A. C., et al. (2019) Integrating Storytelling into a Communication Skills Teaching Program for Medical Oncology Fellows | Descriptive study | The effective practice of medicine requires narrative competence, which is the ability to acknowledge, absorb, interpret, and act on the stories and plights of others. Narrative medicine, which is medicine practiced with narrative competence, improves the well-being of many physicians and their patients and strengthens bonds between them. This study aims to integrate stories into the curriculum as a way of building empathy and warming fellows to the arduous task of dealing with highly emotional content, such as conversations with young patients about transitioning off disease-directed therapy. | The authors adapted the curriculum based on learner feedback and reflection by faculty and teaching assistants and consolidated sessions into quarterly 3–4-hour workshops. | Oncology fellows | Sharing stories can help highly technical learners build reflective ability, mindfulness, and empathy, which are all critical ingredients of the art of medicine. | N/A | N/A |
| 91 | Silva, D. H. (2008) A competency-based communication skills workshop series for pediatric residents | Quantitative study | The Pediatric Residency Program at the UPR School of Medicine needs an advanced communication skills curriculum. The residency program is mostly located at a tertiary care center, which serves most of the population in Puerto Rico. In this context, dealing with complex and difficult situations is a common occurrence for residents and they should be adequately prepared. Thus, a four-unit competency-based workshop series was developed for Pediatrics Residents. | The authors conducted a literature review and needs assessment at the residency program and developed a competency-based, advanced communication skills curriculum. The curriculum contains four units: Communicating Bad News, Communicating in Difficult Physician-Patient situations, Communicating with Adolescents abd Answering Telephone Consultations. The communcating bad news unit was fully developed, implemented and pilot tested. | First year pediatric residents | The intervention group performed significantly better than the non-intervention group in the Pilot Test. Residents found the instructional sessions to be excellent and effective. ` | 9 | N/A |
| 92 | Smith, L., et al. (2013) An educational intervention to improve resident comfort with communication at the end of life | Quantitative study | Several studies have developed didactic modules to teach residents the communication skills necessary for caring for patients at the end of life. Although such modules have demonstrated a measurable impact on resident knowledge and attitudes regarding end-of-life care, none has been widely adopted into resident curricula. Utilizing a strategy of facilitated, case-based, peer interactions, this study sought to assess the feasibility and impact of a novel curriculum in end-of-life education that would be easily incorporated into the preexisting internal medicine resident curriculum. | The study took place at each of the three University of California, San Francisco (UCSF) teaching hospitals, and involved all internal medicine residents at UCSF. The curriculum consisted of two one-hour lunch conference sessions and six one-hour morning reports. A pre- and post-intervention electronic survey was administered. | Internal Medicine residents | The teaching sessions offered in this study were well-attended and well-received by residents. The curriculum impacted resident reports of comfort with specific topics in end-of-life care, including discussions of code status and comfort care. Furthermore, the authors found that this curriculum, although brief, demonstrated a small impact on resident reports of self-efficacy for communication. These findings demonstrate the feasibility of incorporating end-of-life communication skills training into an existing internal medicine resident curriculum. | 9.5 | N/A |
| 93 | Smith, P. E., et al. (2002) Using simulated consultations to develop communications skills for neurology trainees | Mixed methods study | Communication skills are essential for clinical medicine yet, unlike in general practice, trainees in specialist and general medicine are not formally trained in them. | We have used videotaped recording of simulated consultations to evaluate their acceptability and usefulness for training neurology specialist registrars. Twelve specialist registrars in neurology participated; their perceptions of the method were assessed using quanti®ed scales and focus groups. | Neurology registrars | Videotaped consultations with simulated patients are valued by most neurology trainees, both for improving their history-taking skills and for imparting information. The technique could be used more widely in neurology training, and may have a role in assessment. | 9 | 22 |
| 94 | Sullivan, A. M., et al. (2016) The Impact of Resident Training on Communication with Families in the Intensive Care Unit. Resident and Family Outcomes | Prospective study | The aim of the study was to assess the impact of a communication training program on resident skills in communicating with families in an ICU and on family outcomes. | This study conducted a prospective, single-site educational intervention study. Residents attended a 3 session course over 3 weeks which included 2 1-hour didactic sessions and 1 2-hour simulation session which focused solely on more extensive role-playing exercises with feedback. The course content included basic communication skills relevant to meetings or updates with family members in all clinical settings, such as making introductions and establishing an agenda, as well as skills more routinely required in the ICU, such as understanding goals of care and responding empathically to emotion. Residents were asked to complete surveys immediately before and after the course and at 2–3 months after course completion. During the second year of the study, additional funding allowed us to add an observation component wherein research staff observed and documented resident communication with family members in the ICU. Family members’ ratings of specific meetings with ICU residents were collected via family surveys and, for a subgroup of participants (n = 20), in-person semistructured interviews. | Internal medicine residents on ICU rotation | At our institution, an on-site communication training program designed for integration into medical residency programs was associated with strongly positive family member outcomes and significant improvements in residents’ perceived skills. This intervention may serve to prepare residents for optimal communications with patients and family members in ICUs and elsewhere. | N/A | N/A |
| 95 | Szmuilowicz, E., et al. (2010) Improving residents' end-of-life communication skills with a short retreat: a randomized controlled trial | Quantitative study | Internal medicine residents are largely unprepared to carry out end-of-life (EOL) conversations. There is evidence that these skills can be taught, but data from randomized controlled trials are lacking. This paper studied whether a day-long communication skills training retreat would lead to enhanced performance of and confidence with specific EOL conversations. It also studied the effect of the retreat on residents’ ability to respond to patient emotions. | PGY-2 resident volunteers were randomly assigned to a retreat group or a control group. The retreat involved a combination of teaching styles and skills practice with standardized patients. All participants completed questionnaires and were evaluated carrying out two types of conversations (breaking bad news or discussing direction of care) with a standardized patient before (T1) and after (T2) the intervention phase. Conversations were audio-taped and later rated by a researcher blinded to group assignment and time of assessment. | Internal Medicine residents | A short course for residents can significantly improve specific elements of resident EOL conversation performance, including the ability to respond to emotional cues. | 16 | N/A |
| 96 | Tobler, K., Grant, E. and Marczinski, C. (2014) Evaluation of the impact of a simulation-enhanced breaking bad news workshop in pediatrics | Quantitative study | Breaking bad news (BBN) to a family in pediatrics can be extremely challenging for physicians, with the emotional stress of the event and the potential for long-lasting impact on a family adding to the level of difficulty and stress experienced by trainees and practicing physicians when faced with this task. The goal of this study was to develop and evaluate the effectiveness of a simulation-based workshop for teaching pediatric trainees’ communication skills in breaking bad news. | A simulation-based workshop was developed to teach skills in breaking bad news. After a classroom-based introduction, small groups of residents participated in 3 scenarios, each starting with a simulated resuscitation, followed by 2 conversations with the patient’s parent, played by actors. | Residents of the general pediatrics and pediatric emergency medicine programs | Breaking bad news (BBN) to a family in pediatrics can be extremely challenging for physicians, with the emotional stress of the event4 and the potential for long-lasting impact on a family adding to the level of difficulty and stress experienced by trainees and practicing physicians when faced with this task. | 11.5 | N/A |
| 97 | Trickey, A. W., et al. (2017) Two-Year Experience Implementing a Curriculum to Improve Residents' Patient-Centered Communication Skills | Quantitative study | Surgeons must demonstrate effective patient-centered communication to deliver high-quality care. Surgical procedures are often associated with high risks of morbidity and mortality, and surgeons must provide information to help patients make choices after weighing pros and cons of different alternatives. Although surgical patient-reported health outcomes and satisfaction are independently related to perceptions of their surgeons’ empathic abilities, a systematic review revealed that surgeons spend most of patient communica- tion time educating and helping patients make choices, while rarely exploring emotional concerns.  This study describes a 2-year experience implementing a simulation-based curriculum, results of annual communication performance assessments, and resident evaluations. | Eight quarterly modules were conducted on various communication topics. Former patient volunteers served as simulation participants (SP) who completed annual assessments using the Communication Assessment Tool (CAT). During these 2 modules, communication skills were assessed in the following standardized scenarios: (1) delivering bad news to a caregiver of a patient with postoperative intra-cerebral hemorrhage and (2) primary care gallstone referral with contraindications for cholecystectomy. SP-CAT ratings were evaluated for correlations by individual and associations with trainee and SP characteristics. Surgical patient experience surveys are evaluated during the curriculum. | Surgical residents | The simulation-based SP-CAT has shown initial evidence of usability, content validity, relationships to observed communication behaviors and residents skills confidence. Evaluations of different scenarios may not be correlated for individuals over time. The communication curriculum paralleled improvements in patient experience concerning surgeons’ clear explanations. An ongoing surgery resident communication curriculum has numerous educational, assessment, and institutional benefits. | 14 | N/A |
| 98 | Trickey, A. W., et al. (2016) Assessment of Surgery Residents' Interpersonal Communication Skills: Validation Evidence for the Communication Assessment Tool in a Simulation Environment | Quantitative study | Although development of trainees' competency in interpersonal communication is essential to high-quality patient-centered surgical care, nontechnical skills present assessment challenges for residency program directors. The Communication Assessment Tool (CAT) demonstrated internal reliability and content validity for general surgery residents, though the tool has not yet been applied in simulation. The study provides validation evidence for using the CAT to assess surgical residents' interpersonal communication skills in simulation scenarios. | Simulations of delivering bad news were completed by 21 general surgery residents during a mandatory communication curriculum. Upon completion of the 10-minute scenario, standardised participants (SPs) assessed performance using the 14-item CAT rating scale and individually provided feedback to residents. Discrete communication behaviour were recorded on video review by a trained blinded observer. The traits emotional intelligence questionnaire short form (TEI-Que-SF) was completed by the residents 6 months later. SP-CAT ratings are evaluated with respect to learner characteristics, observed behaviors, and TEIQue results. | General surgery residents | The CAT demonstrated content validity in a simulation environment with former patients acting as SPs. This study provides validation evidence relating the SP-CAT to discrete observations of communication behaviours by a trained, reliable observer as well as residents' self-reported emotional intelligence traits. | 13 | N/A |
| 99 | Turner, J. W., et al. (2019) Resident reflections on resident-patient communication during family medicine clinic visits | Qualitative study | A meta synthesis of 48 qualitative studies argued that communication training is particularly important to consider when doctors practice in low-income areas where the doctor and the patient may be operating on very different levels of literacy and within unique cultural experiences. Residency programs emphasize effective doctor and patient interaction. However, training can be time intensive and logistically challenging. This paper examines a blog providing resident peer feedback and an opportunity to explore how residents think about patient communication. | A grounded theory approach examined peer commenting on doctor patient interactions. Between 2012–2015, at a U.S. East Coast Family Medicine Clinic, 35 family medicine residents were recorded interacting with patients, producing a total of 84 videos which were posted to a blog. Residents reflected on these videos resulting in 356 responses, 3162 meaning units and 211 codes. Codes were grouped into 10 themes. Further analysis explored how residents signaled positive and negative doctor communications-related behavior. | Family medicine residents | Residents view their communication with patients as more of a transmission of critical information than an opportunity for dialogue. | N/A | 17 |
| 100 | Ungar, L., et al. (2002) Breaking bad news: structured training for family medicine residents | Quantitative study | Previous research has shown that physicians experience incompetence and difficulty in dealing with patients' feelings after they have broken bad news to them. During the past 10 years, the authors have implemented a longitudinal training program targeting these issues. The present article describes this training and discusses its contribution to doctors' skills at approaching distressed patients. | Each session encompassed the areas of crisis intervention, communication techniques, awareness of their personal attitudes and emotional reactions when breaking bad news, as well as the most prominent issues arising when breaking bad news. | Family medicine residents | In a 1-5 Likert scale, the course received an overall score of 4.47 (SD 0.51). Participants noted that they had gained relevant communication skills for future patient encounters. | 8 | N/A |
| 101 | Watling, C. J. and Brown, J. B. (2007) Education research: communication skills for neurology residents: structured teaching and reflective practice | Mixed methods study | Surveys consistently show that patients want better communication from their physicians. Unfortunately, patient–physician communication remains inadequate much of the time. Time and experience alone often fail to produce improvements in physicians’ communication skills, but there is compelling evidence for a positive effect of communication skills training. To date, the area of communication skills training has been largely neglected in the neurologic literature. The objectives of this pilot project were to develop a communication skills training program specifically for neurology residents, directed at some of the most challenging clinical scenarios that they will face in practice, and to foster reflective practice as a tool for enhancement of communication skills. | A group of 12 neurology residents participated in a series of six case-based communication skills workshops. Each workshop focused on a particular clinical scenario, including breaking bad news, discussing do-not-resuscitate orders, communicating with “difficult” patients, disclosing medical errors, obtaining informed consent for neurologic tests and procedures, and discussing life-and-death decisions with families of critically ill patients. Residents also kept reflective portfolios in which real examples of these interactions were recorded. | Neurology residents | The program was well accepted, and residents rated the workshops as effective and relevant to their practice. Analysis of residents’ portfolios revealed three themes relevant to patient–physician communication: 1) communication is more successful when adequate time is allowed, 2) the ability to empathize with patients and their families is essential to successful interactions, and 3) the development of specific approaches to challenging scenarios can facilitate effective interactions. The portfolios also demonstrated that residents would engage in reflective practice. | 8.5 | 15 |
| 102 | Weissmann, P. F. (2006) Teaching Advanced Interviewing Skills to Residents: A Curriculum for Institutions with Limited Resources | Descriptive study | This paper describes a time-efficient seminar series in communication skills for first-year internal medicine residents, which has been received well by faculty and learners as evidenced by post-seminar surveys and focus groups. | Four seminars, each three hours long, are conducted with the overall curricular goal of teaching internal medicine residents to interview patients effectively and empathically, using a proper balance of patient- and doctor-centered techniques, mirroring Cohen-Cole’s three-function model. | First year Internal Medicine residents | External validation of residents’ performance that improves as a result of the interviewing course described here would be the first validation of a short, relatively time-efficient educational intervention in the domain of communication skills training. | N/A | N/A |
| 103 | Williams, D. M., et al. (2011) Development and evaluation of a program to strengthen first year residents' proficiency in leading end-of-life discussions | Quantitative study | Multiple interventions have been developed to teach and improve internal medicine residents’ end-of-life communication skills, but have not been easily adaptable to other institutions. The purpose of this study was to develop and evaluate a program to enhance physicians’ end-of-life communication with families of dying patients using a format that could be incorporated into an existing curriculum for first-year internal medicine residents. | An end-of-life educational program was developed and evaluated in the context of educating first-year residents at an urban academic medical center during the 2008-2009 academic year. The program consisted of three sessions including an interactive workshop flanked by pre- and post-workshop evaluations in simulated encounter and clinical vignette formats. Simulated encounters were recorded on video and residents’ performances were rated by two independent observers using a 23 point checklist. | First year Internal Medicine residents | Complete data were available for 24 (73%) of 33 residents who participated in the program. The residents’ checklist scores increased significantly from a mean of 48.1 at baseline to 73.9 at follow-up. The increase in the scores on the clinical vignettes was also statistically significant, but of lesser magnitude. | 14.5 | N/A |
| 104 | Wong, R. Y., et al. (2009) Using television shows to teach communication skills in internal medicine residency | Quantitative study | So far, no formal structured teaching methods of the Kalamazoo model exist in the literature. This study intended to reinforce the importance of effective communication with particular attention to the Kalamazoo model by means of cinemeducation. This study aimed to address evidence-based effective communication skills in the formal academic half day curriculum of the authors' core internal medicine residency program, through designing and delivering an interactive session using excerpts taken from medically-themed television shows. | The authors selected two excerpts from the television show House, and one from Gray's Anatomy and featured them in conjunction with a brief didactic presentation of the Kalamazoo consensus statement on doctor-patient communication. To assess the efficacy of this approach a set of standardized questions were given to our residents once at the beginning and once at the completion of the session. | Internal Medicine residents | The residents indicated that their understanding of an evidence-based model of effective communication such as the Kalamazoo model, and their comfort levels in applying such model in clinical practice increased significantly. Furthermore, residents' understanding levels of the seven essential competencies listed in the Kalamazoo model also improved significantly. Finally, the residents reported that their comfort levels in three challenging clinical scenarios presented to them improved significantly. | 11 | N/A |
| 105 | Wood, J., et al. (2004) Patient, faculty, and self-assessment of radiology resident performance: a 360-degree method of measuring professionalism and interpersonal/communication skills | Mixed methods study | This study aimed to develop and test the reliability, validity, and feasibility of a 360-degree evaluation to measure radiology resident competence in professionalism and interpersonal/communication skills. | An evaluation form with 10 Likert-type items related to professionalism and interpersonal/communication skills was completed by a resident, supervising radiologist and patient after resident-patient interactions related to breast biopsy procedures. Residents were also evaluated by faculty, using an end-of-rotation global rating form. Residents, faculty, and technologists were queried regarding their reaction to the assessments after a 7-month period. | Radiology residents | Results from this pilot study suggest that self, faculty, and patient evaluations of resident performance constitutes a valid and reliable assessment of resident competence. Additional data are needed to determine whether the 360-degree assessment should be incorporated into residency programs and how frequently the assessment should be performed. Requiring only a specified number of assessments per rotation would make the process less burdensome for residents and faculty. | 14 | 15 |
| 106 | Wouda, J. C. and van de Wiel, H. B. (2014) The effects of self-assessment and supervisor feedback on residents' patient-education competency using videoed outpatient consultations | Quantitative study | Nowadays, direct observation followed by effective feedback is considered to be a powerful means to teach communication skills in clinical practice. Communication assessment and feedback have already featured in the training of general practitioners and primary care physicians for several decades as part of vocational training and certification. Workplace-based assessment of medical-specialist trainees’ communication occurs less frequently. Furthermore, research into the effects of workplace-based assessment on clinical performance remains underdeveloped. This study aimed to determine the effects of residents’ communication self assessment and supervisor feedback on residents’ communication-competency awareness, on their patient-education competency, and on their patients’ opinion. | The program consisted of the implementation of a communication self-assessment and feedback process using videoed outpatient consultations (video-CAF). Residents wrote down communication learning objectives during the instruction and after each video-CAF session. Residents’ patient-education competency was assessed by trained raters, using the CELI instrument. Participating patients completed a questionnaire about the contact with their physician. | Residents | Forty-four residents and 21 supervisors participated in 87 video-CAF sessions. After their first video-CAF session, residents wrote down more learning objectives addressing their control and rapport skills and their listening skills. Video-CAF participation improved residents’ patient-education competency, but only in their control and rapport skills. Video-CAF participation had no effect on patients’ opinion. | 14 | N/A |
| 107 | Yakhforoshha, A., et al. (2019) Effectiveness of Integrating Simulation with Art-Based Teaching Strategies on Oncology Fellows' Performance Regarding Breaking Bad News | Quantitative study | The task of breaking bad news (BBN) may be improved by incorporating simulation with art-based teaching methods. The aim of the present study was to assess the effect of an integrating simulation with art-based teaching strategies, on fellows’ performance regarding BBN, in Iran. | The study was carried out using quasi-experimental methods, interrupted time series. | Oncology fellows | The results showed that using an integrating simulation with art-based teaching strategies may help oncology fellows to improve their communication skills in different facets of BBN performance. | 15 | N/A |
| 108 | Young, O. M. and Parviainen, K. (2014) Training obstetrics and gynecology residents to be effective communicators in the era of the 80-hour workweek: a pilot study | Quantitative study | To ensure optimal patient care, physicians must establish effective patient-physician relationships and thoughtfully incorporate their patients’ perspectives into their counseling. Historically, these skills are acquired with increasing clinical experience. However, given increasing work-hour restrictions, obstetrics and gynecology residents have fewer opportunities to develop these skills. The objective of this study was to determine if an interactive learning method is an effective tool by which to teach OB/GYN residents how to communicate with complicated patients. | An experiential simulation model was developed to teach OB/GYN residents effective communication skills for dealing with patients experiencing a pregnancy-related complication. A simulated patient interaction was designed for first-year residents. Specific scenarios were constructed based on challenging clinical scenarios identified by second-year residents. Non-judgmental communication, culture competency awareness and reflective listening were key skills that were taught as part of the clinical scenarios. Both acceptability and utility of the exercise with the first-years was assessed by a follow-up survey. | First year obstetrics and gynecology residents | Seven first-year residents participated in the education session consisting of four physician-patient interactions with specific learning objectives for each. These first-year residents all indicated that they would employ the skills practiced during the intervention into their future practice of medicine, and that their comfort level in caring for complex obstetric patients had increased. Moreover, all first-year residents endorsed that this educational strategy was potentially applicable to other aspects of their training. | 8.5 | N/A |
| 109 | Yudkowsky, R., Alseidi, A. and Cintron, J. (2004) Beyond fulfilling the core competencies: an objective structured clinical examination to assess communication and interpersonal skills in a surgical residency | Mixed methods study | The Accreditation Council for Graduate Medical Education (ACGME) has challenged program directors to assess their residents’ core competencies, including communication and interpersonal skills (CIS). This study reports the authors' institution’s experience using a series of standardized patient encounters in an objective structured clinical examination (OSCE) to evaluate CIS in surgical residents. | Standardized patients rated the residents’ ability to maintain a patient-centered approach across 6 challenging communication tasks. Residents received verbal feedback from the patients after each encounter and completed a survey indicating their experience and comfort with each task. Individual and group reports documented resident competency and provided aggregate information for curriculum review. Formal grades were not assigned. | Surgical residents | The standardized patient-based OSCE is an effective method to assess communication and interpersonal skills and provides useful information for curriculum review. | 14.5 | 11 |
